# Sexual dimorphism and the role of estrogen in the immune microenvironment of liver metastases

**DOI:** 10.1038/s41467-019-13571-x

**Published:** 2019-12-17

**Authors:** Simon Milette, Masakazu Hashimoto, Stephanie Perrino, Shu Qi, Michely Chen, Boram Ham, Ni Wang, Roman Istomine, Andrew M. Lowy, Ciriaco A. Piccirillo, Pnina Brodt

**Affiliations:** 1 Department of Medicine, McGill University, Montreal, QC Canada; 2 Cancer Research Program, Research Institute of the McGill University Health Centre, Glen Site, 1001 Décarie Blvd, Montréal, QC H4A 3J1 Canada; 3 Department of Microbiology and Immunology, McGill University, Montréal, Québec, H3A2B4 Canada; 4 Program in Infectious Diseases and Immunology in Global Health, Centre for Translational Biology, Montréal, Québec, H4A 3J1 Canada; 5 Research Institute of the McGill University Health Centre, Montréal, Québec, H4A 3J1 Canada; 6 Centre of Excellence in Translational Immunology (CETI), Montréal, Québec, H4A 3J1 Canada; 7 Division of Surgical Oncology, Department of Surgery, Moores Cancer Centre at UC San Diego Health, 3855Health Sciences Dr., La Jolla, CA 92037 USA; 8 Program in Infectious Disease and Immunity in Global Health, Research Institute of the McGill University Health Centre, Glen Site, 1001 Décarie Blvd, Montréal, QC H4A 3J1 Canada; 9 Department of Surgery, McGill University, Montreal, QC Canada; 10 Department of Oncology, McGill University, Montreal, QC Canada

**Keywords:** Translational research, Metastasis

## Abstract

Liver metastases (LM) remain a major cause of cancer-associated death and a clinical challenge. Here we explore a sexual dimorphism observed in the regulation of the tumor immune microenvironment (TIME) of LM, wherein the accumulation of myeloid-derived suppressor cells (MDSC) and regulatory T cells in colon and lung carcinoma LM is TNFR2-dependent in female, but not in male mice. In ovariectomized mice, a marked reduction is observed in colorectal, lung and pancreatic carcinoma LM that is reversible by estradiol reconstitution. This is associated with reduced liver MDSC accumulation, increased interferon-gamma (IFN-γ) and granzyme B production in CD8^+^ T cells and reduced TNFR2, IDO2, TDO and Serpin B9 expression levels. Treatment with tamoxifen increases liver cytotoxic T cell accumulation and reduces colon cancer LM. The results identify estrogen as a regulator of a pro-metastatic immune microenvironment in the liver and a potential target in the management of liver metastatic disease.

## Abstract (web summary)

It is known that estrogens can affect the immune response to cancer. Here, the authors show that estrogen depletion through ovariectomy reduces the extent of liver metastasis and that this is accompanied by a decrease in the number and function of immunosuppressive cells in the metastatic microenvironment.

## Introduction

We recently identified the TNF receptor 2 (TNFR2) as a key regulator of the immunosuppressive microenvironment (ME) of colon and lung carcinoma liver metastasis (LM)^1^. In TNFR2-null female mice, the incidence of LM was significantly reduced and this was associated with a significant decrease in the accumulation of myeloid-derived suppressor cells (MDSC) and CD4^+^CD25^+^Foxp3^+^ regulatory T cells (Treg) in the liver, as compared to wild-type (WT) mice. This reduction was due, at least in part, to increased MDSC apoptosis in TNFR2-null female mice.

Sex has been recognized as an important biological determinant in the immune response to infection, in autoimmunity and in susceptibility to diseases associated with impaired immune functions, such as cancer. Numerous epidemiological studies have documented naturally occurring sex disparities in the incidence of autoimmune diseases, which are generally more prevalent in females^2–4^, and in the incidence of cancers of nonreproductive organs, which tend to have a male bias^5–10^. These sex disparities in the pathogenesis and manifestations of disease are likely regulated, at least in part, by the respective sex hormones and the underlying changes in gene expression they induce in response to pathological stimuli. However, the mechanisms whereby sex differences affect disease outcome appear to be multifactorial and complex, and remain incompletely understood.

The estrogens, that together constitute the main female sex hormones, are pleiotropic steroids that can influence diverse biological processes spanning development, metabolism, and immunity. Estrogens regulate gene expression via the estrogen receptors (ERs) and chromatin-modifying cofactors to activate specific DNA response elements^11,12^. Two nuclear ERs have been identified: the ERα (encoded by the *Esr1* gene) and the ERβ (encoded by the *Esr2* gene). These receptors bind estrogen with similar affinities, but their tissue distributions are distinct^13,14^. Expression of *Esr1* was documented in most immune cells and their progenitors^15^, including B and T lymphocytes, macrophages, natural killer cells (NK), and dendritic cells (DCs), rendering them particularly responsive to regulation by estrogens^16^. For example, estrogens have been implicated in regulating neutrophil numbers, chemotaxis, and proliferation^17^. Estrogens were shown to regulate DC differentiation^18^ and exert bipotential effects on human macrophages, with low concentrations promoting proinflammatory cytokine production (i.e., IL-1, IL-6, and TNF) and high concentrations blocking their secretion (reviewed in refs. ^10,19^). At physiological levels, estradiol (E2), the major estrogen produced by the ovaries, was reported to drive the differentiation of naive CD4^+^CD25^+^ murine T lymphocytes into immunosuppressive Treg^20,21^. Finally, it was recently demonstrated that 17β-E2, by promoting the secretion of TNF-α, contributes to the accumulation of MDSC in the blood^22^. Together, these studies identify the ER/E2 axis as a key determinant of the immune response to cancer. However, because the role of estrogen signaling is context dependent, its effect on the tumor immune microenvironment (TIME) can vary, depending on the organ site.

The organ sites of cancer metastases and the patient’s sex have emerged as biological factors that can influence the outcome of immunotherapy. Recent results of clinical trials with the PD-1 inhibitor pembrolizumab have revealed that in melanoma and lung cancer patients, the presence of liver (but not lung) metastases predicted a poorer response, suggesting that the immunological status of the liver may have systemic consequences^23,24^. LM in lung cancer patients also predicted a poorer response to the anti-PD-L1 antibody durvalumab^25^. Moreover, similarly to LM, female sex was identified as one of 5 variables with significant association to the response to pembrolizumab^24^. A recent meta-analysis of 20 randomized controlled trials of immune checkpoint inhibitors (ipilimumab, tremelimumab, nivolumab, or pembrolizumab) showed that male patients have a greater treatment benefit from these drugs when compared to control treatments than do female patients^26^. Understanding the factors that regulate the immune ME of LM can therefore have implications, not only for controlling liver metastatic disease but also for optimizing the systemic benefits of immunotherapy.

Here we show that unlike our findings in female mice, neither the number of LM nor MDSC accumulation are altered in TNFR2-null male mice as compared to WT controls and identify estrogen as a major regulator of a prometastatic immune microenvironment in the liver.

## Results

### The role of TNFR2 in liver metastases is sex dependent

**Fig. 1:**
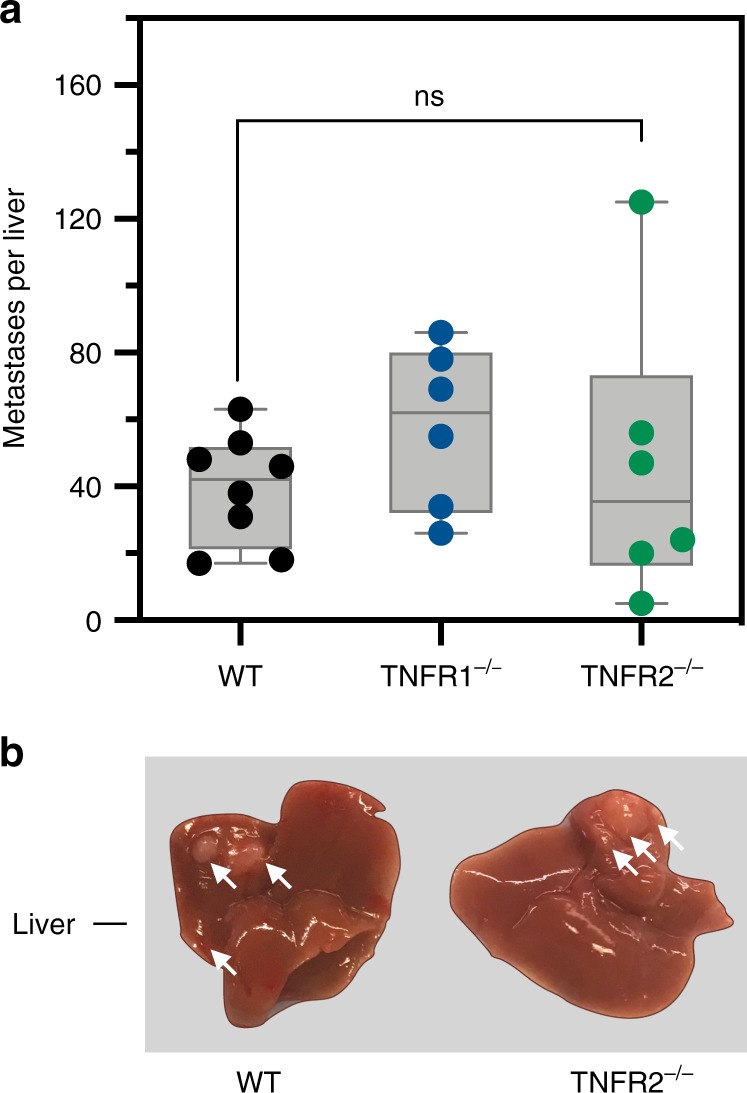
Loss of TNFR2 expression does not reduce LM in male mice. Male Bl6 mice were inoculated with 5 × 10^4^ MC-38 cells via the intrasplenic/portal route. Liver metastases were enumerated 20 days later. Shown in **a** are pooled data of two experiments and in **b** representative livers from tumor-injected mice (*n* = 8 for WT, *n* = 6 for TNFR1^−/−^, and *n*=6 for TNFR2^−/−^ mice; horizontal bars denote median values). NS, not significant as determined by the Mann–Whitney test. Box and whiskers graphs: the box extends from the 25th to 75th percentiles, the middle line denotes the median and the whiskers extends from the minimum to the maximum value.

We previously reported that in female mice, TNFR2 plays a critical role in colorectal LM by regulating MDSC and Treg accumulation in the liver^1^. However, intriguingly, when LM was evaluated in male mice, following inoculation of age-matched TNFR2-null mice of the same cohort with colon carcinoma MC-38 cells via the intrasplenic/portal route, we found that the numbers of hepatic metastases in these mice did not significantly differ from those in WT controls. Similarly, to our results in female mice, no difference in LM was observed between TNFR1^−/−^ and WT mice (Fig. 1a, b).

### In male TNFR2^−/−^ mice liver MDSC accumulation is unaltered

**Fig. 2:**
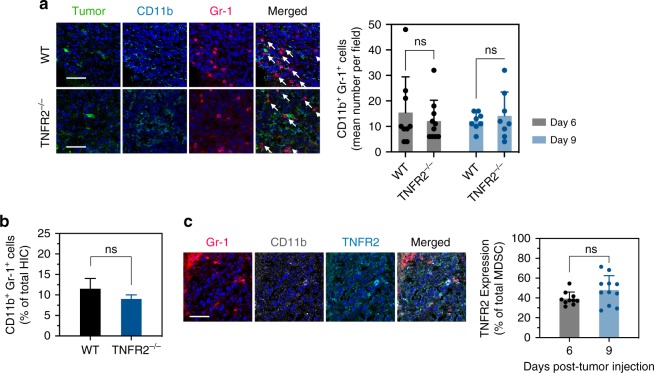
Loss of TNFR2 expression does not affect MDSC accumulation in male mice. Livers were resected at the indicated time intervals postintrasplenic/portal injection of 5 × 10^5^ GFP-tagged MC-38 cells (green) (**a**, **c**) or 5 × 10^4^ MC-38 cells (**b**), 14 μm frozen sections prepared and immunostained with the indicated antibodies followed by Alexa Fluor 568 (red) for Gr-1, and Alexa Fluor 647 (cyan) or Alexa Fluor 700 (white) for CD11b and Alexa Fluor 647 (cyan) for TNFR2. Shown in **a** (left) are representative images of 20 fields acquired for each experimental group (*n* = 3, day 9) and in **a** (right) the mean numbers (±s.e.m.) of CD11b^+^Gr-1^+^ cells enumerated in each group at the indicated time intervals following tumor injection. Shown in **b** are results of FC performed on immune cells isolated 9 days post tumor injection and immunostained with the indicated antibodies. They are expressed as means (±s.e.m.) of three separate analyses, each performed on livers obtained from six mice per group. Shown in **c** (left) are representative images of TNFR2^+^CD11b^+^Gr-1^+^ cells visualized in 30 fields in sections derived from male mice inoculated with MC-38 cells 9 days earlier and in **c** (right) the results expressed as % TNFR2^+^ cells (±s.e.m.) of total CD11b^+^Gr-1^+^ cells counted per field in sections derived at the indicated time intervals following tumor injection. NS, not significant as determined by the a two-tailed Student's *t*-test. Scale bars correspond to 100 µm on histology images.

In female TNFR2^−/−^ mice, we previously observed a significant reduction in MDSC accumulation in and around LM, relative to controls^1^. We therefore next compared the immune cell infiltrate in WT and TNFR2^−/−^ male mice, following intrasplenic/portal inoculation of MC-38 cells, using flow cytometry (FC) and immunofluorescence (IF). IF revealed the presence of CD11b^+^Gr-1^+^ cells around sites of tumor infiltration in both WT and TNFR2^−/−^ male mice. Unlike our findings in the female mice, however, and consistent with the lack of difference in the incidence of LM, the numbers of CD11b^+^Gr-1^+^ cells detected in the two groups were similar (Fig. 2a), and this was confirmed by FC (Fig. 2b) that was performed on isolated hepatic immune cells (HIC). Further characterization of these cells showed that in male WT mice, the proportion of CD11b^+^Gr-1^+^ cells expressing TNFR2 (30–40%; Fig. 2c) was significantly lower than that in female mice (75–90%)^1,27^.

### The liver TIME of male and female mice are distinct

To further explore possible differences in the TIME of male and female mice, we first compared the proportional accumulation of HIC in the livers of tumor-bearing male and female mice 7 days post inoculation of MC-38 cells via the intrasplenic/portal route. FC revealed that the total number of HIC collected from male mice was 1.68-fold higher than that from female mice, possibly due to the proportionally larger volume of male livers (Supplementary Fig. 1a). Immunophenotyping of the HIC revealed that the average proportion per liver of G-MDSC (CD11b^+^Ly6G^High^Ly6C^Mid^) but not of M-MDSC (CD11b^+^Ly6C^High^Ly6G^Mid^) was 4-fold higher in male than in female mice (Supplementary Fig. 1b) and in addition, the expression of TNFR2 on female MDSC was significantly (3-fold) higher than on male MDSC (Supplementary Fig. 1c), consistent with the differential effect of TNFR2 loss on the accumulation of these cells in the liver. In contrast, no marked differences were observed in the average numbers of T lymphocytes in the livers of male and female mice, although the number of NK cells increased in the female livers (Supplementary Fig. 1d). These data suggested that myeloid cell recruitment to the TIME of LM in male and female mice may be differentially regulated.

### Ovariectomy reduces LM in a site-specific manner

**Fig. 3:**
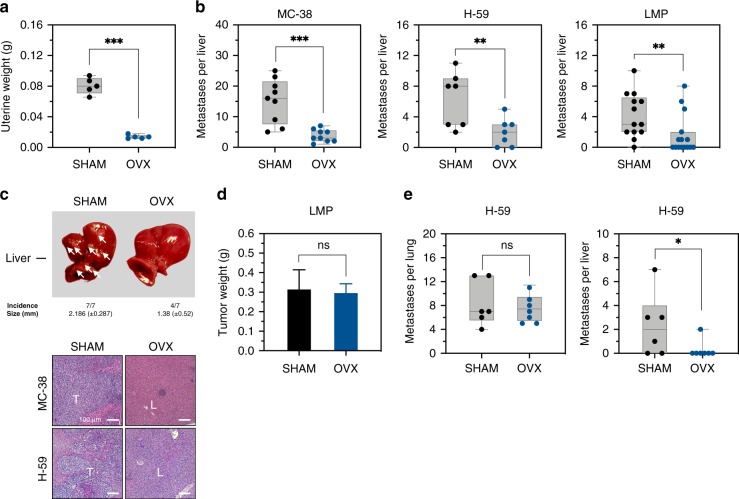
Ovariectomy reduces LM in a site-specific manner. Experimental LM were generated by inoculation of 2 × 10^5^ colon carcinoma MC-38 or lung carcinoma H-59 cells via the intrasplenic/portal route, spontaneous LM of PDAC were generated by intrapancreatic injection of 1 × 10^6^ Kras^G12D/+^;LSL-Trp53^R172H/+^;Pdx-1-Cre (LMP) cells and experimental lung metastases by intravenous inoculation of 2 × 10^5^ H-59 cells. Mice were sacrificed and metastases enumerated 15–30 days later. Shown in **a** are the uterine weights (means ± s.e.m.) as measured 3 weeks post ovariectomy or sham surgery. Shown in **b** are the numbers of visible metastases counted per liver of individual mice inoculated with the indicated tumor cells (*n* = 10 for MC-38 injected; *n* = 7 for H59 injected; and *n* = 14 for LMP-injected mice). Representative livers (top) and H&E stained liver sections (bottom) from MC-38-inoculated mice are shown in **c** and pancreatic tumor weights of LMP-injected mice (mean ± s.e.m., *n* = 14) are shown in **d**. Shown in **e** are the numbers of experimental lung (left) and liver (right) metastases counted 15 days following a tail vein injection of 2 × 10^5^ H-59 cells (left panel: *n* = 5 for sham and *n* = 7 for OVX mice, right panel, *n* = 6 for sham and *n* = 7 for OVX mice). Horizontal bars denote median values. *−*p* ≤ 0.05; **−*p* ≤ 0.01; ***−*p* ≤ 0.001; ****−*p* ≤ 0.0001; NS, not significant as determined by the Mann–Whitney test (metastases) or Student's *t*-test (organ weights). Box and whiskers graphs: the box extends from the 25th to 75th percentiles, the middle line denotes the median and the whiskers extends from the minimum to the maximum value. Scale bars correspond to 100 µm on IF images.

Having observed a sexual dimorphism in the control of MDSC accumulation and LM, we next investigated the role that hormonal regulation plays in controlling the immune microenvironment associated with LM. To this end, female C57BL/6 mice were surgically ovariectomized (OVX) as means of reducing circulating estrogen levels. Reduced uterine weight, indicative of decreased physiological sex hormone levels, was confirmed 3 weeks post ovariectomy (Fig. 3a). Experimental LM were then generated in the OVX and sham-operated control mice by injecting MC-38 or (lung carcinoma) H-59 cells via the intrasplenic/portal route. In addition, spontaneous metastases of pancreatic ductal adenocarcinoma (PDAC) were generated by intrapancreatic injection of 10^6^ LMP cells^28^. These tumor models were selected because they are highly metastatic to the liver, represent histological tumor types that are sex hormone independent and metastasize to the liver during the clinical course of the disease. Mice were sacrificed and LM enumerated 15–30 days later. In all three groups, we observed a significant reduction in the number of hepatic metastases in OVX mice, as compared to sham-operated controls (Fig. 3b). Hematoxylin and eosin (H&E)-stained formalin-fixed paraffin-embedded liver sections confirmed the decrease in the number and size of LM in OVX mice (Fig. 3c). Importantly, no difference in the growth of the primary, intrapancreatic tumors was observed in LMP-implanted animals, as reflected by similar tumor weights in all mice (Fig. 3d). Together, these data suggested that ovarian sex hormones promoted colon, lung, and pancreatic carcinoma LM in the female mice. To determine whether this decrease in growth of metastases was site-specific, we tail vein-injected OVX and control mice with 2 × 10^5^ H-59 cells to generate experimental lung and LM. When lung metastases were enumerated 21 days later, no difference was observed between the estrogen-deprived and control mice, while the number of LM in the same OVX mice was significantly decreased relative to controls (Fig. 3e), identifying a site selectivity in the hormonal control of metastases. Finally, ovariectomy did not reduce the incidence of experimental LM in TNFR2^−/−^ mice (Supplementary Fig. 2a) neither was the number of lung metastases reduced in TNFR2^−/−^ mice (Supplementary Fig. 2b), suggesting that the tumor growth-promoting effect of ovarian hormones in the liver may be linked to TNFR2 signaling.

The ovaries are a source of several female sex steroid hormones, including E2 and progesterone. To more specifically assess the role of β-E2, the most abundant estrogen in the ovaries, we subcutaneously implanted OVX mice with silastic capsules containing 17β-E2, 1 week prior to the inoculation of MC-38 cells, and analyzed LM 15 days post tumor inoculation. An enzyme-linked immunosorbent assay (ELISA) confirmed that the serum E2 concentrations, while below detection levels in most OVX mice, were restored to the normal range in OVX mice implanted with β-E2-containing capsules (Fig. 4a). We found that reconstitution of E2 restored the number of liver metastases to control levels (Fig. 4b), identifying it as the estrogen most critical for metastatic outgrowth in the liver.

**Figure:**
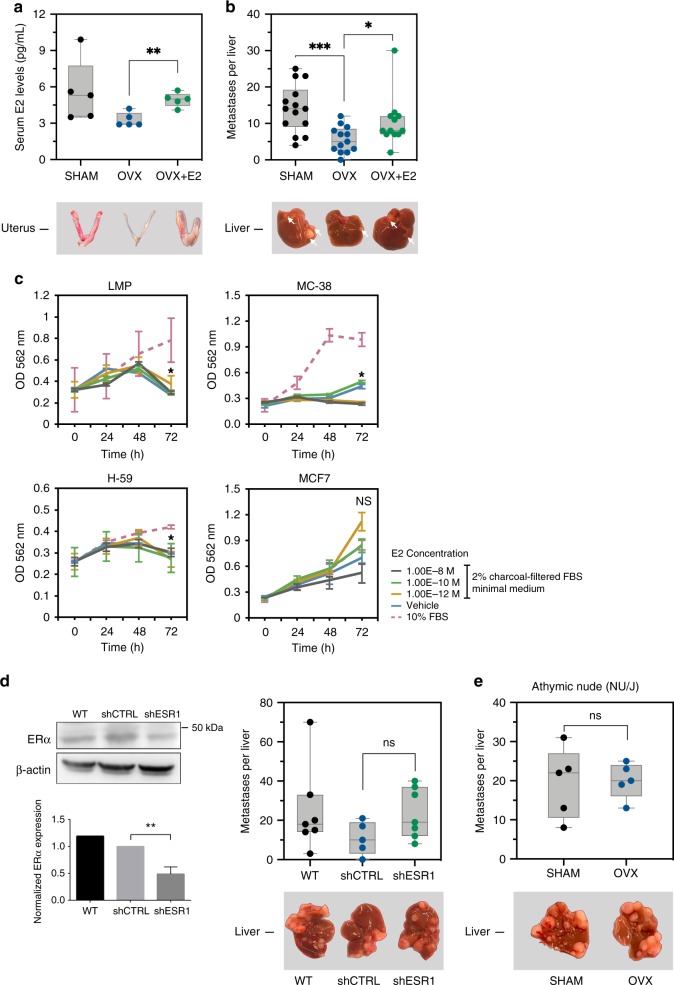
Fig. 4. Reduced LM in OVX mice is reversed by β-E2 and is T cell-dependent. Shown in **a** (top) are E2 levels in the serum of Sham, OVX, and β-E2-reconstituted OVX (OVX + E2) mice as measured by ELISA, and in **a** (bottom) representative uteri isolated from the respective experimental groups (*n* = 5). Shown in **b** are the numbers of visible metastases counted per liver in sham operated, OVX, and OVX + E2 mice (*n* = 15 for sham; *n* = 13 for OVX; and *n* = 12 for OVX + E2 mice; horizontal bars denote median values). Shown in **c** are the results of MTT assays performed on the indicated cells using the E2 concentrations shown. Control cells were treated with vehicle-only. Shown are mean OD ± s.d. of three experiments performed per cell type (statistical analysis was used to compare 10^−12^M E2 to 10% FBS). Shown in **d** (left) are representative immunoblots of MC-38 cells stably transfected with a lentivirus expressing *Esr1* shRNA (shESR1) or a scrambled sequence (shCTRL) and the mean normalized expression of *Esr1* (±s.d.) as compared to beta-actin (*n* = 3; a complete gel image can be seen in Supplementary Fig. 8). Shown in **d** (right) are the numbers of visible metastases counted per liver in mice injected with 2 × 10^5^ MC-38^WT^ cells (WT), mock-transfected MC-38 cells (shCTRL) or ESR1-knocked down MC-38 cells (shESR1; *n* = 7 for WT; *n* = 5 for shCTRL; and *n* = 7 for shESR1 groups; horizontal bars denote median values). Shown in **e** are the numbers of visible metastases counted per liver in sham operated, OVX, and OVX + E2 athymic nude (NU/NU) mice (*n* = 5; horizontal bars denote median values). *−*p* ≤ 0.05; **−*p* ≤ 0.01 ***−*p* ≤ 0.001; NS, not significant as determined by the Mann–Whitney test (metastases) or Student's *t*-test (ELISA, MTT assay, and Western blot). Box and whiskers graphs: the box extends from the 25th to 75th percentiles, the middle line denotes the median and the whiskers extends from the minimum to the maximum value.

To rule out a direct effect of estrogen on tumor cell proliferation, we performed an methylthiazolyl-diphenyl-tetrazolium bromide (MTT) assay. Tumor cells were incubated for 24 h in phenol red-free DMEM medium containing low (2%; charcoal-filtered) FBS, then treated with β-E2 at concentrations ranging from 10^−8^–10^−12^ M. When compared to control cells (cultured in 2% FBS only), no marked effect of β-E2 on MC-38, LMP or H-59 cell proliferation was observed at any of the concentrations used, or at any time point (24, 48, and 72 h) tested, although, under similar conditions, β-E2 did increase the proliferation of the estrogen-dependent MCF-7 cells, that were used as a positive control, to levels similar to 10% FBS complete medium (Fig. 4c). Furthermore, when we injected MC-38 cells in which expression of the ~50 kDa ERα isoform was stably reduced using lentivirus expressing *Esr1* short hairpin RNA (shRNA; Fig. 4d) into estrogen-competent female mice, we did not observe significant reductions in the numbers of LM, as compared to WT MC-38 cells or cells transfected with a nonsilencing control sequence (Fig. 4d). Finally, when MC-38 cells were injected into athymic nude mice that were OVX prior to tumor cell injection, no significant difference in the number or size of LM in these mice, as compared to sham-operated controls was observed (Fig. 4e), suggesting that the effect of ovariectomy was dependent on an intact T cell immune response. Collectively, these data strongly suggested that reduced metastases in estrogen-deprived female mice was due mainly to effects on the immune microenvironment associated with the metastases and not to direct effects on the tumor cells. This was as also suggested by the aggressive phenotype of these cancer cells in male mice and by the liver-specific effect of OVX on tumor cell growth in our models.

### Estrogen regulates a tumor-permissive tumor immune microenvironment (TIME) in the liver

**Fig. 5:**
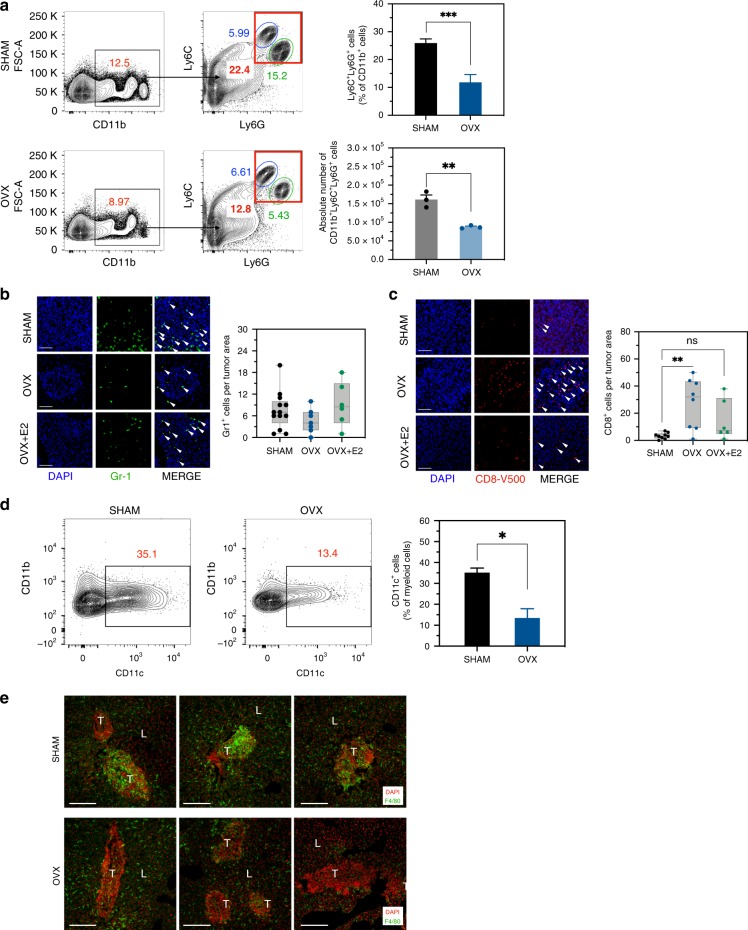
Estrogen promotes an immunosuppressive microenvironment in the liver. FC was performed on immune cells isolated 7 days post intrasplenic/portal injection of 5 × 10^5^ MC-38 cells and immunostained with the indicated antibodies. Shown in **a** (left) are the contour plots obtained for each group and in **a** (right) the proportion (%) of CD11b^+^Ly6C^+^Ly6G^+^ cells per total liver immune cells expressed as means (±s.d.) of three experiments, each performed on a pool of five livers per group. Shown in **b**, **c** and **e** are representative confocal images from 10 μm cryostat liver sections obtained 7 days post intrasplenic/portal inoculation of 5 × 10^5^ MC-38 cells and immunostained with the indicated antibodies followed by Alexa Fluor 568 (green) for Gr-1 (**b**), Alexa Fluor 647 (red) for CD8 (**d**), Alexa Fluor 647 (green) for F4/80 (**e**), and 4′,6-diamidino-2-phenylindole (DAPI; blue in **b** and **c**, and red in **e**). Shown on the right of the confocal images are the numbers (means ± s.e.m.) of the indicated cells per field counted in 7 fields (OVX, OVX + E2) or 12 fields (sham) per group. Shown in **d** (left) are representative flow cytometric contour plots obtained for the indicated groups and in **d** (right) the mean proportion of CD11b^+^CD11c^+^ cells per liver based on a pool of five livers per group, analyzed in triplicates. *−*p* ≤ 0.05; **−*p* ≤ 0.01; ***−*p* ≤ 0.001; NS, not significant as determined by the Student's *t*-test. Box and whiskers graphs: the box extends from the 25th to 75th percentiles, the middle line denotes the median and the whiskers extends from the minimum to the maximum value. Scale bars correspond to 100 µm on IF images.

MDSCs represent a heterogeneous population of CD11b^+^Ly6C^+^Ly6G^+^ myeloid cells recruited to sites of cancer growth, where they exert immunosuppressive functions, including suppression of CD8^+^ cytotoxic T cell (CTL) and Treg recruitment^29^. Because our data identified a sex disparity in the control of these cells in the TIME of the liver, we analyzed whether the observed reduction in LM in OVX mice was due to an impaired recruitment of MDSC to the TIME. OVX and control mice were euthanized 7 days post MC-38 inoculation, and HIC were isolated and analyzed by FC and quantitative real-time reverse transcription polymerase chain reaction (qRT-PCR). Analysis of gene expression first confirmed that tumor-infiltrating T cells and MDSCs expressed ERα (Supplementary Fig. 3). We found a 2-fold decrease in the accumulation of CD11b^+^Ly6C^+^Ly6G^+^ in the livers of OVX mice, as compared to sham-operated controls (Fig. 5a), which was due mainly to a marked reduction in the G-MDSC (CD11b^+^Ly6C^Mid^Ly6G^High^) population. IF performed on cryostat liver sections, obtained from the same mice confirmed the presence of these cells in the margins of micrometastases in all groups and showed a trend toward reduced accumulation in OVX mice (Fig. 5b). This corresponded to an increase in the number of CD8^+^ T cells within the same margins (Fig. 5c, see additional data below). No marked difference in the accumulation of NK cells was observed (Supplementary Fig. 4), but we did observe a reduction in the accumulation of CD11c^+^ cells in estrogen-deprived mice (Fig. 5d). Moreover, a marked decrease in tumor-associated F4/80^+^ macrophages, that are known to play an important role in LM progression^30^, was observed in the livers of OVX mice (Fig. 5e).

To determine whether these changes in the immune response were tumor cell specific, we injected OVX mice with H-59 lung carcinoma (Fig. 6a) and LMP PDAC cells via the intrasplenic/portal route (Fig. 6b), and analyzed the composition of the TIME by FC. Similarly to MC-38-injected mice, H-59- and LMP-injected OVX mice showed a reduction in the accumulation of liver MDSC, that could be reversed upon estrogen replacement (Fig. 6a, b), although the reduction in LMP-injected mice, while consistent and statistically significant was not as marked as that observed in MC-38-injected mice. Taken together, these data identified estrogen as an important and multifunctional regulator of the TIME of liver metastatic disease. Having observed that the growth of LMP cells in the pancreas and lung metastasis of H-59 cells were not altered in OVX mice, we also undertook to compare the TIME in these organs in estrogen-competent and deprived mice. In OVX mice implanted into the pancreas with LMP cells, there was no significant difference in the accumulation of G-MDSC and CD8^+^ CTL in the pancreatic tumors, as compared to tumors in sham-operated or estrogen-restored mice (Fig. 6c). Moreover, in contrast to the liver TIME, we found in the pancreas, a negligible accumulation of Ly6C^High^ M-MDSC. In the lungs of mice intravenously injected with H-59 cells, no change in the accumulation of CD8^+^ CTL was observed in response to estrogen deprivation (Fig. 6d). In addition, the accumulation of lung M-MDSC was not significantly affected by OVX, while there was a negligible accumulation of G-MDSC in this organ. Together, these findings revealed organ-specific differences in myeloid cell accumulation in the lungs, pancreas, and livers of tumor-bearing mice. They suggested, moreover, that estrogen deprivation had a unique and site-specific effect on the TIME of the liver.

**Figure:**
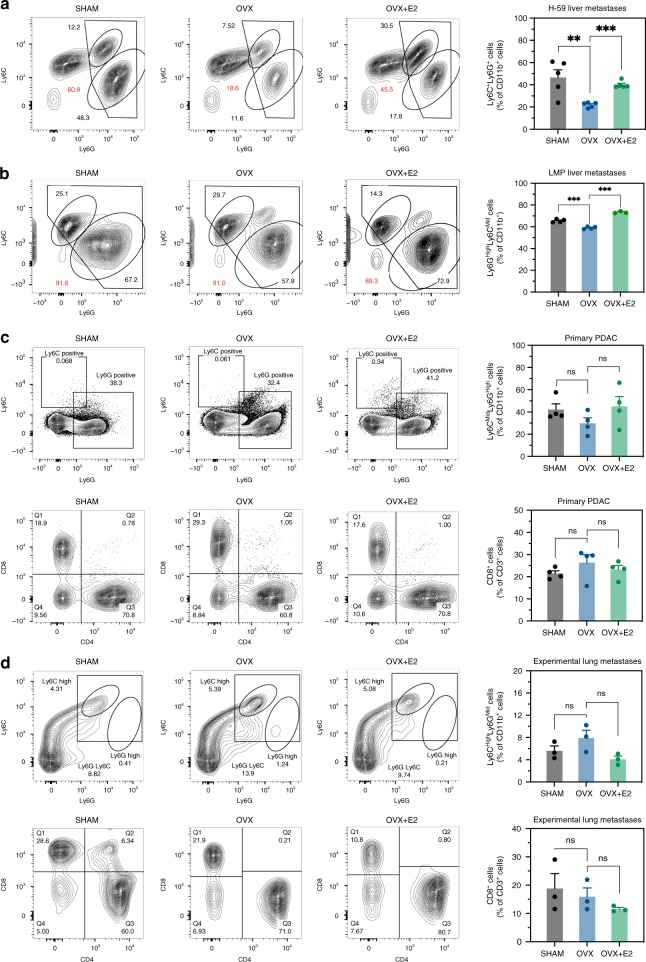
Fig. 6. The effect of estrogen deprivation on the TIME is liver specific. FC was performed on immune cells isolated 7 days post intrasplenic/portal injection of 5 × 10^5^ H-59 cells (**a**) or LMP cells (**b**) and immunostained with the indicated antibodies. Orthotopic PDAC tumors were generated by intrapancreatic injections of 1 × 10^6^ LMP cells (**c**), and experimental lung metastases were generated by intravenous injections (tail vein) of 5 × 10^5^ H-59 cells (**d**). Shown in **a**, **b** (left) are representative flow cytometric contour plots obtained with each of the indicated immune cells populations (for complete gating strategy, please see Supplementary Fig. 9) and in the bar graphs **a**, **b** (right) the mean proportions (%; ±s.e.m.) of MDSC per liver based on four mice per group, analyzed individually. Shown in **c** (left) are representative flow cytometric contour plots obtained with immune cells derived from pancreatic LMP tumors that were first gated for CD45^+^ cells and in **d** (left) from experimental lung metastases of H-59 cells that were gated based on viability and size. Shown in the bar graphs **c**, **d** (right) are the mean proportions (%; ±s.e.m.) of the indicated immune cells (*n* = 3). *−*p* ≤ 0.05; **−*p* ≤ 0.01; ***−*p* ≤ 0.001; NS, not significant as determined by the Student's *t*-test.

### Estrogen regulates immunoregulatory genes in the liver

**Fig. 7:**
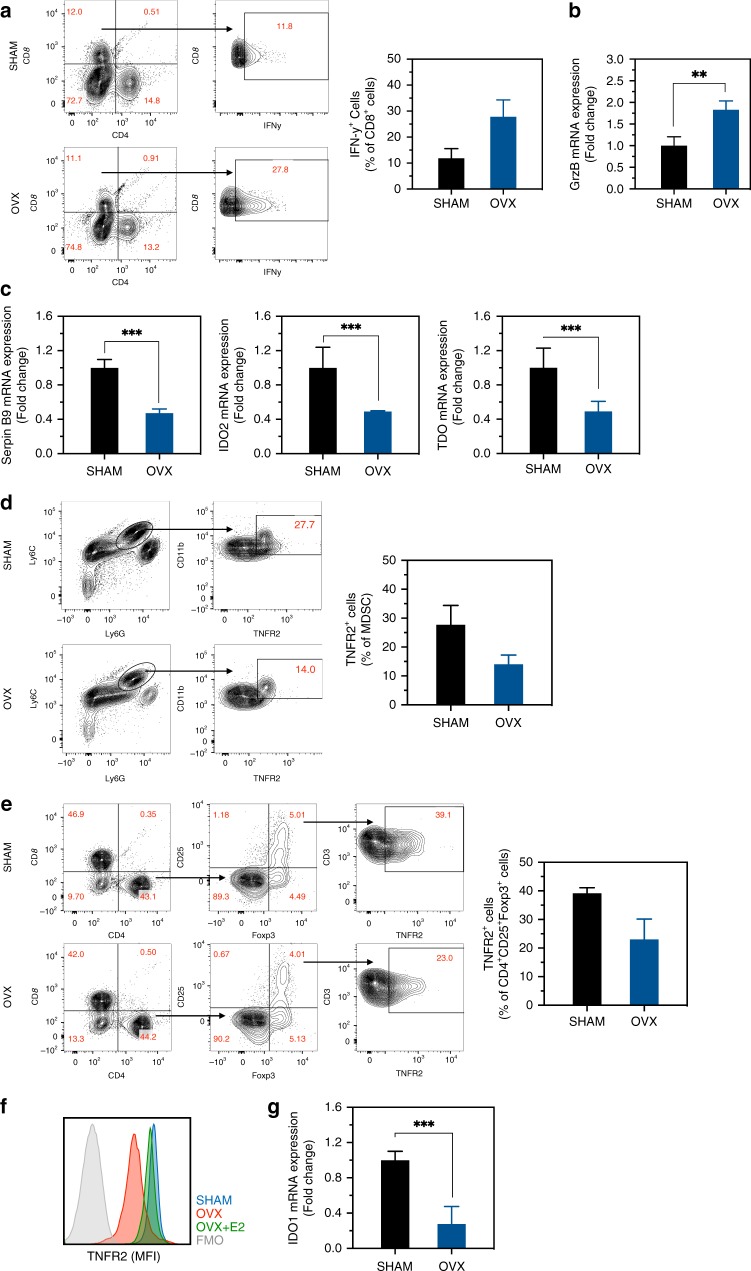
Estrogen regulates the expression of immunosuppressive genes in the liver. FC was performed on immune cells isolated 7 days post intrasplenic/portal injection of 5 × 10^5^ MC-38 cells and immunostained with the indicated antibodies. Shown in **a** (left) are results of an IFN-γ production assay performed on liver-derived CD8^+^ T cells extracted from OVX or sham-operated mice and in **a** (right) the mean proportions (%) of CD8^+^IFN-γ^+^ cells per liver (±s.e.m.) based on a pool of five livers per group, analyzed in triplicates. Shown in **b** are results of qRT-PCR (±s.d.) performed on RNA extracted from CD8^+^ T cells sorted from livers of tumor-injected mice (data normalized to GAPDH; *n* = 3). Shown in **c** are results of qRT-PCR performed on whole liver RNA obtained from tumor-bearing sham or OVX mice. The results for each of the indicated transcripts are based on livers obtained from three mice and expressed as means (±s.d.) relative to sham operated mice that were assigned a value of 1, all normalized to GAPDH. Shown in **d**, **e** are representative flow cytometric contour plots obtained with each of the indicated immune cells populations (left) and in the bar graphs (right) the relative frequency of TNFR2^+^ cells per liver, pooled from five livers per group and analyzed in duplicates. Shown in **f** are mean fluorescence intensity (MFI) plots (for TNFR2) obtained for the same CD4^+^CD25^+^Foxp3^+^ cells analyzed in **e**. Shown in **g** are results of qRT-PCR expressed as means (±s.d.) performed on RNA extracted from liver-derived CD4^+^Foxp3^+^ T cells isolated by cell sorting (data normalized to GAPDH; *n* = 3). *–*p* ≤ 0.05; **–*p* ≤ 0.01; ***–*p*≤ 0.001; NS, not significant as determined by the Student's *t*-test. Box and whiskers graphs: the box extends from the 25th to 75th percentiles, the middle line denotes the median and the whiskers extends from the minimum to the maximum value.

Activated CD8^+^ T lymphocytes are major mediators of the adaptive antitumor immune response and characteristically produce increased interferon-gamma (IFN-γ) levels^31^. To determine whether estrogen affects T cell activation and cytokine production in the liver, we measured IFN-γ levels in CD3^+^CD8^+^ T cells isolated from livers of MC-38-inoculated OVX and sham control mice following an ex vivo stimulation with PMA and ionomycin. Flow cytometric analyses revealed a 2-fold increase in IFN-γ production in T cells isolated from OVX mice, as compared to controls (Fig. 7a), suggesting a potentiated CTLs immune response. Increased granzyme B (GrzB) mRNA expression in these cells (Fig. 7b) confirmed FC findings. Because the number of Foxp3^+^ cells was not significantly altered by estrogen deprivation (as shown in Fig. 7e), this proportional increase in the accumulation of activated T cells could result in a more effective antitumor immune response in the livers of these mice.

Serpin B9 (an inhibitor of GrzB), indolamine-2,3-dioxygenase (IDO), and tryptophan-2,3-dioxygenase (TDO; tryptophan catabolic enzymes) are immunosuppressive proteins that are highly induced by elevated estrogen levels during pregnancy to protect developing fetuses from maternal alloreactive T cells^32,33^. These proteins can also be expressed by hepatocytes and tumor-associated DCs and were implicated in immune tolerance to malignant cells^34–36^. We measured their expression levels in the liver using qRT-PCR and observed a >2-fold reduction in Serpin B9, IDO2, and TDO expression in OVX mice (Fig. 7c), suggesting that estrogen was involved in regulating their expression in the liver.

We identified TNFR2 as a regulator of immunosuppression (i.e., MDSC survival and function^1^) in the livers of female, but not male mice. We asked, therefore, whether TNFR2 expression itself, and thereby the response to TNF-α could be estrogen regulated. Tumor-infiltrating MDSCs and Tregs were isolated and immunophenotyped. We found that TNFR2 expression was significantly reduced in monocytic (M)-MDSCs (CD11b^+^Ly6C^High^Ly6G^Mid^) and in CD4^+^CD25^+^Foxp3^+^ cells isolated from OVX mice, as compared to controls (Fig. 7d–f), and this could account for reduced MDSC accumulation, as we have previously shown^1^, and increased T cell activation in estrogen-depleted mice. Indeed, splenocytes collected from WT female mice and treated ex vivo with 10^−7^ M β-E2 had a 3.5-fold increase in TNFR2 mRNA expression, as compared to vehicle-treated controls (Supplementary Fig. 5). We also found that LM-infiltrating Treg cells from OVX mice had a significantly reduced IDO1 expression (Fig. 7g), confirming a deregulated function.

### Estrogen regulates myeloid cell function and tumor cell proliferation in the liver TIME

**Fig. 8:**
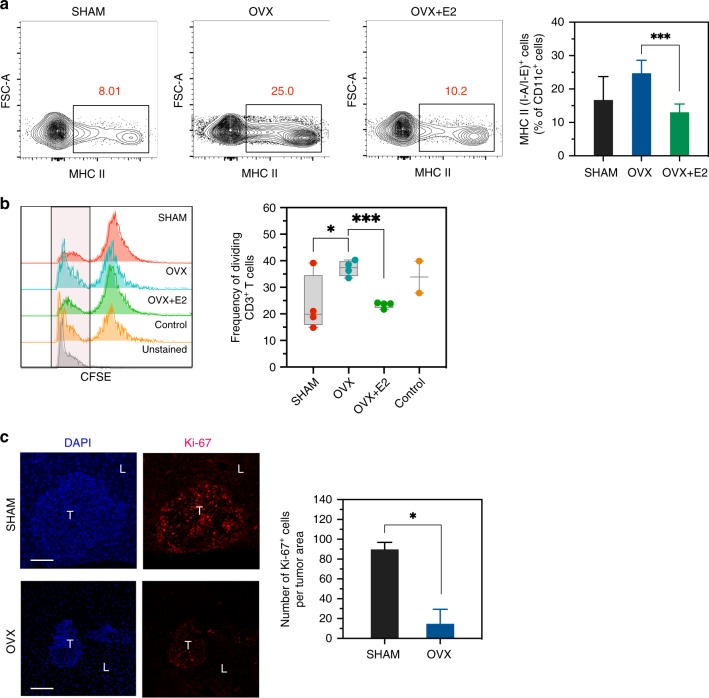
Estrogen deprivation alters myeloid cell function in the liver TIME. FC was performed on immune cells isolated 7 days post intrasplenic/portal injection of 5 × 10^5^ MC-38 cells and immunostained with the indicated antibodies. Shown in **a** are representative flow cytometric contour plots obtained with each of the indicated immune cell populations (left) and in the bar graph (right) the relative frequency of CD11c^+^MHC II (I-A/I-E)^+^ cells per liver, based on a pool of five livers per group analyzed in triplicates. Shown in **b** (right) are representative results of a T cell suppression assay using liver-derived MDSC from sham, OVX, and OVX + E2 mice and in **b** (left) individual results obtained with each of the replicates per group (horizontal bars denote means). Shown in **c** are representative confocal images from sections of livers immunostained with the indicated antibodies followed by Alexa Fluor 568 (red) for Ki-67 and 4′,6-diamidino-2-phenylindole (DAPI; blue, left) and the number of Ki-67^+^ cells per tumor area (right; means ± s.e.m.) of the indicated cells per field counted in 10 fields. *−*p* ≤ 0.05; **-−*p* ≤ 0.01; ***−*p* ≤ 0.001; NS, not significant as determined by the Student's *t*-test. Box and whiskers graphs: the box extends from the 25th to 75th percentiles, the middle line denotes the median and the whiskers extends from the minimum to the maximum value. Scale bars correspond to 100 µm on IF images.

Finally, we found that estrogen-depleted (but not estrogen-reconstituted) OVX mice had increased DC activation (Fig. 8a), consistent with a more robust antitumor immunity. Moreover, to determine whether ovariectomy also affected the function of liver-derived MDSC, thereby controlling T cell expansion, we performed a suppression assay using splenic CD3^+^ T cells as targets. We found the T cells which were coincubated with MDSC derived from livers of OVX mice had a superior proliferative capacity, as compared to T cells incubated with MDSC harvested from sham or OVX + E2 livers (Fig. 8b). This confirmed the immunosuppressive function of liver-derived MDSC and indicated that estrogen deprivation altered not only the mobilization of MDSCs into the liver, but also their suppressive function. Importantly, to determine if these combined effects decreased the ability of metastatic cells to proliferate in the liver, we measured Ki-67 expression in the tumor cells. IF performed on liver sections of tumor-injected OVX and sham-operated mice revealed a marked decrease in Ki-67 positive tumor cells in OVX mice (Fig. 8c). Collectively, these results strongly implicated estrogen in the regulation of multiple factors that can contribute to an immunosuppressive TIME in the liver, including TNFR2 expression.

### Tamoxifen inhibits the outgrowth of colon carcinoma LM

**Fig. 9:**
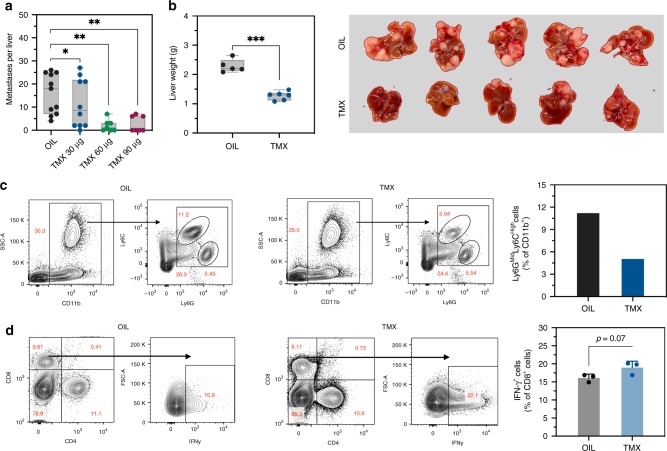
Tamoxifen inhibits metastatic expansion in the liver in a dose-dependent manner. Experimental LM were generated by inoculation of 2 × 10^5^ MC-38 colon carcinoma cells via the intrasplenic/portal route. Subcutaneous injections of tamoxifen or oil were administered on alternate days for 16 days. Mice were sacrificed 21 days post tumor inoculation and LM enumerated. Shown in **a** are numbers of visible metastases counted per liver (*n* = 10; horizontal bars denote median values). Shown in **b** are liver weights (left) of mice treated with 90 µg TMX on alternate days (*n* = 8), and representative livers (right). Shown in **c** are representative flow cytometric contour plots of HIC isolated 7 days post injection of 5 × 10^5^ MC-38 cells from mice treated with 60 µg TMX (or vehicle) on alternate days and stained with the indicated antibodies. Shown in **d** are results of an IFN-γ production assay performed in triplicates on CD8^+^ T cells extracted from mice treated with 60 µg TMX (or vehicle) on alternate days for 7 days. They are based on a pool of five livers per group.*−*p* ≤ 0.05; **−*p* ≤ 0.01, as determined by the Mann–Whitney test (metastases) or Student's *t*-test (organ weights, FC). Box and whiskers graphs: the box extends from the 25th to 75th percentiles, the middle line denotes the median and the whiskers extend from the minimum to the maximum value.

Selective estrogen receptor modulators (SERM) represent a class of molecules that act on the ER to enhance or inhibit its activity. Tamoxifen (TMX), an ER inhibitor of this class is routinely used in the treatment and prevention of hormone-dependent malignancies, including ER-positive breast cancer. To determine whether tamoxifen could recapitulate the effect of ovariectomy on LM, we injected mice with MC-38 cells and this was followed with three weekly subcutaneous injections of 4-hydroxytamoxifen or oil used as vehicle control. Mice were sacrificed and LM enumerated 21 days later. In TMX-treated animals, we observed a significant and dose-dependent reduction in the number of LM, as compared to controls (Fig. 9a, b). Similarly to results in OVX mice, we found in TMX-treated mice a reduction in the average number of liver MDSC (Fig. 9c) and a corresponding 2-fold increase in IFN-γ production levels in CTLs extracted from their livers (Fig. 9d), indicative of an enhanced antitumor T cell immunity in these mice. These data suggest that FDA-approved ER inhibitors could have translational application in the management of liver metastatic disease.

## Discussion

**Fig. 10:**
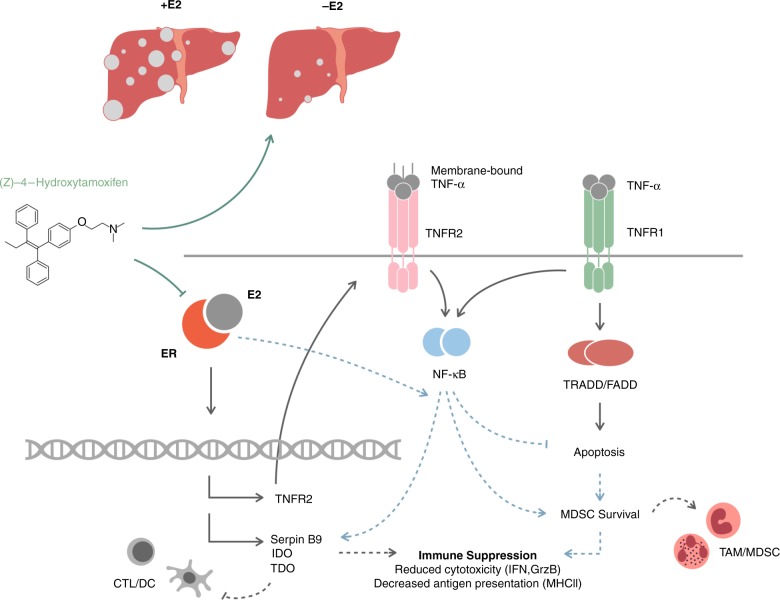
The multifaceted role of estrogen in regulating an immunosuppressive TIME in the liver. Shown is a diagrammatic representation of postulated mechanisms that may be underlying the observed estrogen-mediated regulation of an immunosuppressive TIME in the liver. The ER/E2 signaling pathway can promote metastatic expansion in the liver by inducing TNFR2 transcription. In turn, TNFR2 may rescue immune cells from TNFR1-mediated death signals triggered by the TNF-α-rich microenvironment of LM and/or initiate signal transduction, leading to transcriptional activation of immunosuppressive gene products. This favors MDSC survival and suppressive activity, and reduces CD8^+^T-cell-mediated antitumor responses. ER signaling (possibly in conjunction with, or independently of TNFR2) also regulates transcription of immunosuppressive proteins in the liver to enhance immune evasion. Tamoxifen treatment blocks the ER/E2 axis and reduces immune tolerance to improve tumor cell clearance (dashed lines indicate postulated molecular pathways).

We observed a significant reduction in the outgrowth of LM in OVX female mice injected with highly aggressive colorectal, pancreatic or lung carcinoma cells. This reduction was liver specific and coincided with a marked reduction in MDSC accumulation and their suppressive activity in this organ. It was also associated with a significant increase in immunocompetent CD8^+^ T cells, and both could be reversed upon E2 replacement. Furthermore, in the livers of OVX mice, we observed a marked reduction in the expression levels of several proteins with immune-modulating activities, including IDO2, TDO, and TNFR2. This corresponded to an increased activity of tumor-infiltrating CTL, indicative of a potentiated antitumor immune response. Taken together, these results identify estrogen as the orchestrator of a multicellular and multifactorial immunosuppressive TIME, permissive to metastatic expansion (see a proposed mechanism(s) of estrogen-induced hepatic immunosuppression in Fig. 10). It should be noted, however, that although collectively our data strongly suggest that the main effect of estrogen deprivation on LM, in our models, was due to an altered TIME, we cannot at present entirely rule out the possibility that other, estrogen-regulated, protumorigenic, and prometastatic mechanisms also contributed to reduced LM in the OVX mice.

In a recent report by Pan and colleagues, the number of peripheral blood M-MDSC was shown to be significantly increased in pregnant, as compared to nonpregnant or postpartum women, and their frequency correlated with serum estrogen and progesterone levels^37^. Importantly, the authors demonstrated that treatment of these M-MDSC in vitro with E2, but not with progesterone enhanced both their expansion and suppressive activity through STAT3 activation. This report implicated estrogen in the regulation of MDSC-mediated immunosuppression during pregnancy, when protection of the fetus from immune rejection is critical. Svoronos et al. using estrogen-insensitive tumor models recently reported that E2-conveyed signals contributed to the mobilization and immunosuppressive activity of CD11b^+^ Gr-1^+^ MDSC in a STAT3-dependent manner^38^. In our mouse models of experimental LM, this mechanism of sex hormone-mediated immunosuppressive cell control may be co-opted by expanding tumors, promoting their escape from immune surveillance. It was indeed reported that MDSC expand in gestational mice, enhancing B16-F10 melanoma cell metastasis by exerting an inhibitory effect on NK cell activity^39^. The latter findings are also consistent with the decreased expression of IDO2 and TDO (both upregulated during pregnancy^40,41^) that we observed in the livers of estrogen-ablated tumor-bearing mice.

The molecular processes underlying reduced MDSC accumulation in the livers of OVX mice are likely complex and remain to be fully elucidated. We have previously shown that TNFR2 is required for MDSC survival, and consistent with other reports^42^, we show here that TNFR2 expression is regulated by estrogen. This suggests that the reduction in LM in estrogen-deprived mice may be mediated, at least partially, through reduced TNFR2 expression in these cells. Interestingly, we observed that TNFR2 expression was significantly reduced in the M-MDSC, although their accumulation was not significantly altered in OVX mice. TNFR2 was shown to be required for the suppressive function of M-MDSC^43^. Our results suggest, therefore, that estrogen deprivation can have a dual effect on the liver TIME, reducing G-MDSC accumulation on one hand, while also affecting the functions of M-MDSC by downregulating TNFR2 expression and thereby, decreasing their suppressive activity (Fig. 8b). ER may regulate TNFR2 expression directly, because a partial ER element has been identified 1 kb upstream of the transcription start site (Supplementary Fig. 6), or it may be interacting with NF-κB to coregulate TNFR2. Indeed, both ERα and ERβ were shown to cross talk with the NF-κB axis through interactions with p50, c-Rel, RelA-p50, and IkB (reviewed in ref. ^44^). It also remains to be determined whether some of the changes observed in the expression of immunosuppressive factors, such as IDO are mediated directly through estrogen signaling or are secondary to changes in TNFR2 expression and signaling or the expression of other, estrogen regulated signaling axes.

TNFR2 may, in fact play a unique and essential role in MDSC accumulation in the liver that is distinct from other effects downstream of estrogen signaling. This unique role may be related to the rapid increase in TNFα production upon tumor cell entry into the liver, as we and others have documented^45–49^ and may contribute to the liver-specific consequences of ovariectomy, noted in this study. In this context, it is of significance that we found very low levels (≤1%) of M-MDSC in the TIME of the pancreas, while the levels of G-MDSC, while higher (38%) showed a minor but insignificant reduction in OVX mice. Similarly, in the lung TIME, neither subpopulation was significantly altered, consistent with the lack of effect of estrogen deprivation on tumor growth in these sites. The liver immune response is uniquely adapted to its function as a metabolic organ and programmed to protect the host from the antigenic overload associated with dietary and commensal bacterial products with inflammatory potential. It is also instrumental in fetal immune tolerance^50,51^. Serpin B9 produced by hepatocytes^52,53^ contributes to the generalized immune tolerance of the liver and consistent with several other studies, we show here that this protein is estrogen regulated^34,54,55^. In addition, IDO1 produced by hepatic stellate cells downstream of STAT3 activation may also contribute to a state of immune unresponsiveness^56,57^. Thus, estrogen deprivation can have unique and organ-specific effects on the immune response of the liver through direct or indirect effects on several immunosuppressive factors. Interestingly, we found that the expression of Serpin B9 was not affected by ovariectomy in TNFR2^−/−^ mice (Supplementary Fig. 7). This suggests that estrogen can indirectly regulate the expression of immunosuppressive enzymes in the liver, possibly through regulation of TNFR2. Further studies using mice with conditional, cell-type specific ERα and ERβ deletions will be required to fully understand the complex and multifaceted role that estrogen can play in regulating the hepatic microenvironment associated with LM and its apparent site specificity.

Previously, we reported that in TNFR2-null female mice injected with MC-38 cells, the accumulation of FoxP3^+^CD25^+^ Treg cells in the liver was also markedly reduced^1^, but intriguingly, we did not observe a reduction in the accumulation of these cells in OVX mice, although they have been functionally altered. This difference in Treg accumulation may be due, at least in part, to differences in TNFR2 expression levels in the two models. Thus, while in TNFR2-null mice, this receptor was totally lacking, in OVX mice, TNFR2 expression, although reduced (42%) was not completely abolished and may have provided the threshold TNFR2 expression levels required for Treg survival in the tumor ME of the liver.

Collectively, our results highlight a hitherto underappreciated sexual dimorphism in the regulation of the immunosuppressive and prometastatic microenvironment of the liver and provide evidence that estrogen plays a complex and multifactorial role in its regulation. They also provide a mechanistic framework for the recent intriguing clinical observations that identified sex and LM as determinants of the success of immunotherapy^23,24^. They therefore provide a rationale for further exploration of the benefits of SERM in the clinical management of hormone-independent cancers with a propensity to metastasize to the liver, such as GI malignancies.

Several MDSC-targeting agents have been shown to improve antitumor immune responses in cancer patients (reviewed in ref. ^58^) and to potentiate the effects of immune checkpoint inhibitors^59^. A recent explorative case study in Sweden on 23,154 patients diagnosed with LM has shown that ~15% of all female patients diagnosed with LM were younger than 50 years old, with breast cancer LM and colorectal cancer LM accounting for 32.4 and 21.2% of LM diagnosed in this age group, respectively^60^. Our findings suggest that in premenopausal female patients (age <50), combining SERM with immunotherapy may be beneficial in preventing or limiting the outgrowth of LM.

## Methods

### Animals

All mouse experiments were carried out in strict accordance with the guidelines of the Canadian Council on Animal Care (CCAC) “Guide to the Care and Use of Experimental Animals” and under the conditions and procedures approved by the Animal Care Committee of McGill University (AUP number: 5260). Mouse experiments were performed in C57BL/6 mice bred in the animal facility of the RI-MUHC (Glen Site). LMP cells were implanted in B6129 F1 mice, the strain of origin for this tumor^28^, that were also bred in the animal facility of the RI-MUHC. Nu/Nu female mice were purchased from Charles River Canada. All mice were used at the ages of 7–10 weeks old. Control mice (WT, sham, and oil-treated) were age matched to experimental mice (OVX, OVX + E2, and TMX-treated).

### Cells

The murine colorectal carcinoma MC-38 cells, and the Lewis lung carcinoma (LLC) subline H-59 cells are syngeneic to the C57BL/6 strain. Their origins and metastatic properties have been described in detail previously^1,61^. MC-38 cells were originally from an NCI repository and were obtained as a kind gift from Dr. Shoshana Yakar (New York University, NY). They were recently authenticated by Didion and colleagues using single nucleotide polymorphism profiling^62^. H-59 is a subline of the LLC that was developed by the Brodt laboratory and maintained as a frozen stock since 1986^63^. The PDAC LMP cell line originated from a LM that arose in the genetically engineered Kras^G12D/+^;LSL-Trp53^R172H/+^;Pdx-1-Cre (KPC) mouse model (*Kras*^*G12D/+*^; *LSL-Trp53*^*R172H*/+^; *Pdx-1*^*Cre*^ mice), as described in detail elsewhere^28^ and maintained in the Lowy laboratory since its derivation. In syngeneic B6.129 F1 mice implanted in the pancreas with LMP cells, tumor growth and metastasis mimic the aggressive clinical behavior of PDAC. All cell lines were routinely tested for common murine pathogens and mycoplasma contamination, as per the McGill University Animal Care Committee and the McGill University Biohazard Committee guidelines. All cells were maintained as a frozen stock and generally cultured in vitro for up to 4 weeks only, prior to use in the in vivo experiments, in order to minimize genetic drifts and changes to their metastatic phenotypes. They were expanded in a humidified incubator at 37 °C with 5% CO_2_ in DMEM medium (Wisent), supplemented with 4 mM L-glutamine, 4.5 gL^−1^ glucose, 100 Uml^−1^ penicillin, and 100 μgml^−1^ streptomycin solution (Sigma); 2 gL^−1^ sodium pyruvate and 10% fetal bovine serum (FBS; Wisent).

### Ovariectomy

Mouse ovariectomy (OVX) was performed according to the McGill University Standard Operating Procedures (SOP N°206.01) and with the approval of the Animal Care Committee of McGill University and the Research Institute of the McGill University Health Center. Briefly, sexually mature female mice aged 7–8 weeks were used for this procedure. Mice were administered carprofen (Rimadyl®; 20 mgkg^−1^; s.c.), 30 min prior to surgery and anaesthetized using isoflurane. Animals were placed in sternal recumbence; their backs shaved and then sterilized using 70% ethanol and a 2% chlorexidin solution. For each ovary, a single 0.5 cm dorsal flank incision was made, penetrating the abdominal cavity. The exposed ovary was removed by cauterization. The incisions in the peritoneal wall were sutured, and incisions in the skin were closed with metal clips. Injection of carprofen (s.c.) provided postoperative analgesia. In control, sham-operated mice, two 0.5 cm dorsal flank incisions penetrating the abdominal cavity were made, but the ovaries were not removed.

### Sex steroid replacement

β-E2 (Sigma) was thoroughly mixed in sterile sesame oil (Sigma) at a concentration of 18–36 μgml^−1^. Placebo capsules were filled with sesame oil only. Capsules were prepared from silastic tubing and plugged with 3 mm wooden applicator sticks. The capsules were filled with the hormone solution, capped, and incubated overnight in the remaining hormone/oil solution to equilibrate, then implanted s.c. in female mice that were OVX 10 days earlier.

### Measurement of serum estrogen levels

Blood was collected from age-matched sham-operated, OVX, and E2-implanted OVX mice, and the serum analyzed by the University of Virginia Center for Research in Reproduction Ligand Assay and Analysis Laboratory, using the validated Calbiotech kit (catalog #ES180S-100) for mouse E2 ELISA testing, as per the manufacturer’s instructions^64^.

### Experimental LM

Experimental LM were generated by intrasplenic/portal injections of 5 × 10^4^ or 2 × 10^5^ tumor cells (as indicated), followed by splenectomy as we previously described^1^. Animals were euthanized 15–20 days later, and visible metastases on the surfaces of the livers were enumerated and sized without prior fixation. Where indicated, fragments of the livers were also fixed in 10% phosphate buffered formalin, paraffin embedded, and 10 μm sections stained with H&E to detect micrometastases and quantify the metastatic burden, as shown.

### Experimental lung metastasis

Experimental lung metastases were generated by intravenous (tail vein) injections of 2 × 10^5^ H-59 cells. Animals were euthanized 15 days later. Lungs were harvested and fixed in Bouin’s solution for 48 h. Following fixation, visible metastases on the surfaces of the lungs were enumerated.

### Spontaneous PDAC LM

Spontaneous PDAC liver metastases were observed following the intrapancreatic implantation of 1 × 10^6^ LMP cells in 25 µL Matrigel (Corning, NY, USA) mixed with 25 µL phosphate buffered saline (PBS) as described^65^. Briefly, B6.129 mice were anesthetized using isoflurane. After local shaving and disinfection, the abdominal cavity was opened by a 1.5-cm longitudinal incision into the left upper quadrant. A volume of 50 µL of ice-cold Matrigel/cancer cell mixture was then slowly injected into the tail of the pancreatic parenchyma using an ice-cold 27-gauge needle and an ice-cold calibrated syringe. The needle was kept in the injection site for 30 s prior to removal to prevent leakage. Animals were euthanized 21–30 days post tumor implantation, at which time metastases were visible on the surface of the liver and were enumerated and sized without prior fixation.

### Immunostaining and confocal microscopy

C57BL/6 female mice were injected via the intrasplenic/portal route with 2 × 10^5^ MC-38 or H-59 cells and the livers perfused at the time intervals indicated, first with PBS and then with 4 ml of a 4% paraformaldehyde solution. The perfused livers were placed in 4% paraformaldehyde for 48 h and then in 30% sucrose for an additional 48 h before they were stored at −80 °C. For immunostaining, 20 μm cryostat sections were prepared, incubated first in a blocking solution (1% bovine serum albumin and 1% FBS in PBS) and then for 1 h each with the primary antibodies, used at the indicated dilutions, and the appropriate Alexa Fluor conjugated secondary antibodies (The antibodies used in this study, their source and the dilutions used are listed in Supplementary Table 1), all at room temperature (RT). The sections were mounted in the Prolong Gold antifade reagent (Molecular Probes, Eugene, Oregon, USA) and confocal images were captured with a Zeiss LSM-780 microscope with a spectrum detection capability. Immunostained cells were quantified blindly in at least 10 images acquired per section, per group.

### Isolation of HIC and FC

To analyze early changes in the TIME, mice were injected with 5 × 10^5^ tumor cells via the intrasplenic/portal route, and the livers removed 3, 7, or 9 days later (as indicated). Liver homogenates were prepared in cold PBS and filtered through a stainless steel mesh using a plunger. The filtrates were centrifuged at 60 G to separate the hepatocytes, the supernatants containing the nonparenchymal cell fraction centrifuged at 480 *g* and the pellets resuspended in 10 ml of a 37.5% Percoll solution in HBSS containing 100 Uml^−1^ heparin and centrifuged at 850 *g* for 30 min to obtain the immune cell-rich fraction. Prior to FC, red blood cells were removed using the ACK (ammonium–chloride–potassium) solution and 1 × 10^6^ cells were immunostained with the indicated antibodies. Data acquisition was with a BD Canto flow cytometer and FACS Diva software and the data analyzed using the FlowJo software. For flow cytometric experiments on hepatic leukocytes, single cells were gated based on size (forward scatter), granularity (side scatter) and viability using an eFluor™ 780 fixable dye (eBioscience™, ThermoFisher).

### Ex vivo T cell activation for cytokine production

Experimental LM were generated by injecting 5 × 10^5^ MC-38 cells via the intrasplenic/portal route. Livers were resected 7 days later and T cells isolated and stimulated for 4 h with phorbol-12-myristate-13-acetate (PMA; 5 ngml^−1^; Sigma, CAT. P-8139) and ionomycin (500 ngml^−1^; Sigma, CAT. I-0634) in the presence of a protein transport inhibitor (BD GolgiStop™, CAT. 554724). The activated T cells were first immunostained for extracellular markers, and then fixed and permeabilized for IFN-γ staining prior to analysis by FC.

### RNA extraction and qPCR

Total cellular RNA was extracted from snap-frozen liver fragments using the Trizol reagent (Life Technologies, Inc., Burlington, Ontario, Canada), according to the manufacturer’s instructions. Two μg of total RNA were reverse transcribed and the cDNA used for qPCR analysisusing the primer sets listed in (Supplementary Table 1).

MTT assay

Tumor cells were plated into 96-well plates at 10,000 cells/well in phenol red-free DMEM medium (Wisent) supplemented with 2% charcoal-filtered, hormone-free FBS (Wisent) and incubated for 24 h, at which time β-E2 was added (or not) at the indicated concentrations and the cells incubated for up to 72 h. At the end of the incubation period, 10 µL of 5 mgml^−1^ MTT (Sigma) were added to each well and cell density analyzed as per the standard procedure.

### ERα silencing by shRNA

The pLKO.1-puro Lentiviral EsRα shRNA and nonmammalian shRNA clones were provided by the Genetic Perturbation Service of the Goodman Cancer Research Centre and Department of Biochemistry of McGill University. Three shRNAs clones were evaluated, and the most effective was used for transfection (clone #R-676-M-1, TRC clone ID:TRCN0000026201, and gene ID:13982- (https://portals.broadinstitute.org/gpp/public/clone/search). In brief, plasmids expressing the shRNA and packaging vectors were introduced into HEK293T cells using the X-tremeGENE transfection reagent (Sigma), according to the manufacturer’s instructions. Virus particles were concentrated from the supernatants and added to MC-38 cells together with 4 μgml^−1^ polybrene. The infected cells were incubated for 8 h, the medium refreshed and the cells cultured for an additional 48–72 h before adding fresh medium containing 10 μgml^−1^ puromycin. Puromycin-resistant clones were selected 2 weeks later and ERα levels assessed by Western blotting.

### Western blotting

Cells were lysed using the RIPA buffer (50 mM Tris-HCl, pH 8, 150 mM NaCl, 0.1%Triton X-100, 0.1% SDS, and 0.5% sodium deoxycholate) supplemented with the Protease Inhibitor Cocktail (Roche cOmplete Mini, Sigma-Aldrich Canada, Oakville, Ontario) for 30 min at 4 °C. Total cell lysates were clarified by centrifugation at 13,000 *g* for 20 min. Proteins were loaded onto 10% polyacrylamide gels under reducing conditions as (20 μg/lane), transferred to a polyvinylidene fluoride membrane and probed first, overnight at 4 °C, with the primary antibodies to ERα diluted 1:500 in tris-buffered saline + Tween 20 containing 5% BSA and then for 20 min at room temperature with a horseradish peroxidase-conjugated secondary antibody diluted 1:1000. Signals were detected using PierceTM ECL plus (ThermoFisher Scientific).

### T cell suppression assay

Splenocytes from naive mice were isolated and red blood cells lysed as described above. Splenic CD3^+^ T cells were sorted by fluorescence-activated cell sorting (FACS), stained with CellTrace™ carboxyfluorescein succinimidyl ester (ThermoFisher Scientific) and incubated for 48 h in RPMI with Dynabeads® Mouse T-Activator CD3/CD28 (ThermoFisher Scientific) in a 96-well plate at 37 °C. Liver-derived MDSC from sham, OVX, and OVX + E2 mice were isolated 7 days post tumor injection and sorted as described above. MDSC were then added to the preactivated splenic T cells at a ratio of 1:1. In the control condition, no MDSC were added to activated T cells. After 48 h of coincubation, T cells were harvested and analysis of CFSE intensity was performed by FC.

### Tamoxifen treatment

Experimental liver metastases were generated by intrasplenic/portal injections of 2 × 10^5^ tumor cells, followed by splenectomy. Mice were inoculated s.c. on alternate days with the indicated concentration of 4-Hydroxitamoxifen (Sigma) in sterile sunflower seed oil (Sigma) or with sunflower seed oil only as control.

### Statistical analyses

The nonparametric Mann–Whitney test was used to analyze all metastasis data and a two-tailed Student's *t*-test was used to analyze ex vivo and in vitro data and the IF results.

### Reporting summary

Further information on research design is available in the Nature Research Reporting Summary linked to this article.

## Supplementary information

Supplementary Information

Reporting Summary

## Data Availability

The authors declare that the data supporting the findings of this study are available within the paper and its supplementary information files. Unprocessed (raw) data can be made available by the corresponding author upon reasonable request.
