# Supplementary material for: Sexual dimorphism and the role of estrogen in the immune microenvironment of liver metastases
Source: Nat Commun. 2019 Dec 17;10:5745. doi: 10.1038/s41467-019-13571-x (PMC6917725; doi:10.1038/s41467-019-13571-x)
Supplement: Supplementary file 1 — Supplementary Information [file 41467_2019_13571_MOESM1_ESM.pdf]

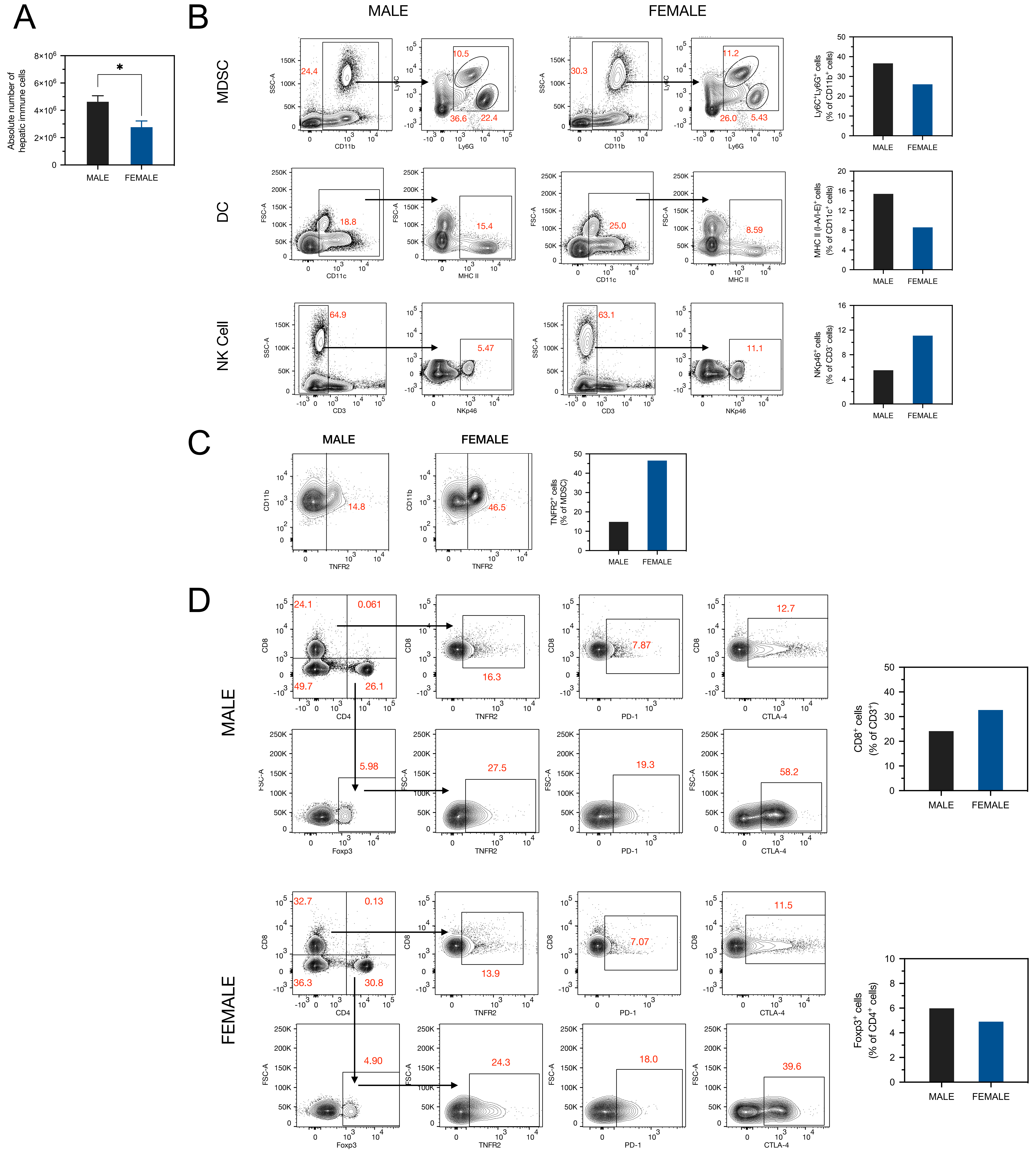

Supplementary Figure 1. The immune microenvironments of liver metastases in male and female mice are distinct
Flow cytometry was performed on immune cells isolated 7 days post intrasplenic/portal injection of 5x10⁵ MC-38 cells and immunostained with the indicated antibodies. Shown in (A) are absolute numbers of hepatic leukocytes (± SEM) isolated from male or female mice (n=5). Shown in (B) are the flow cytometric contour plots (numbers indicate population frequency expressed as % of parental gate) obtained for each of the indicated immune cell populations including $CD11b^+Ly6C^+/Ly6G^+$ cells (B, top), $CD11b^+CD11c^+MHC\ II^+$ cells (B, middle), and $CD3^-NKp46^+$ cells (B, bottom). Shown in (C) are flow cytometric profiles of tumor-derived MDSC and in (D) flow cytometric profiles of tumor-derived ($CD3^+$) T cells, stained with the indicated antibodies. Hepatic immune cells were pooled from 5 mice per group. Summary bar graphs are shown on the right. *-p ≤ 0.05 as determined by the Student T test.

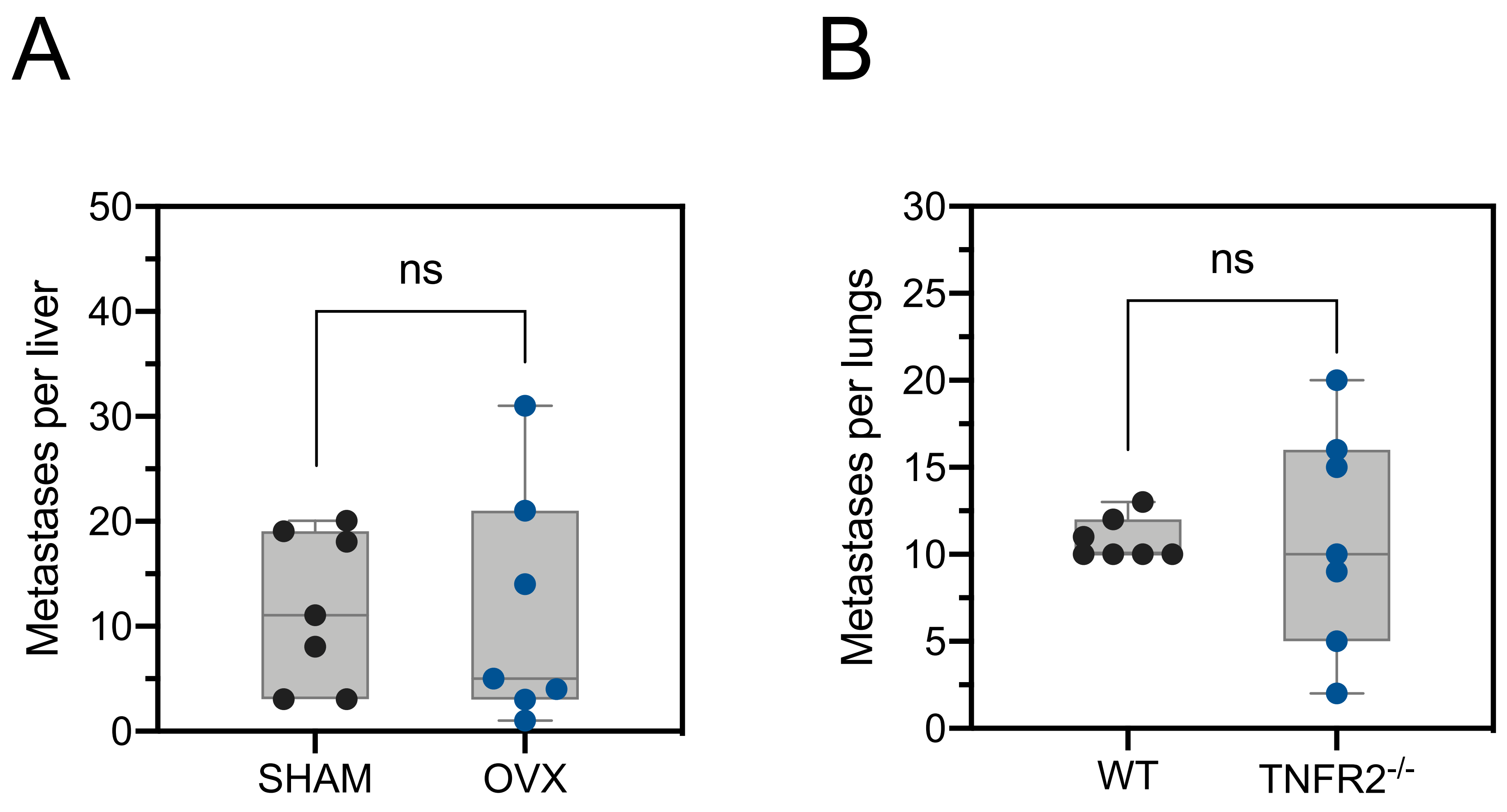

Supplementary Figure 2. Ovariectomy does not reduce the incidence of liver or lung metastases in TNFR2-null mice

Experimental LM were generated by injections of $2x10^5$ MC-38 cells via the intrasplenic/portal route (A) and experimental lung metastases were generated by intravenous injection (tail vein) of $2x10^5$ H-59 cells (B). Mice were sacrificed and visible LM and lung metastases enumerated 15 days later. Shown are the numbers of visible metastases counted per individual livers or lungs of mice in the indicated groups (n=7; horizontal bars denote median values). NS-not significant. Box and whiskers graphs: the box extends from the 25th to 75th percentiles, the middle line denotes de median and the whiskers extends from the minimum to the maximum value. NS-not significant as determined by the Mann-Whitney test.

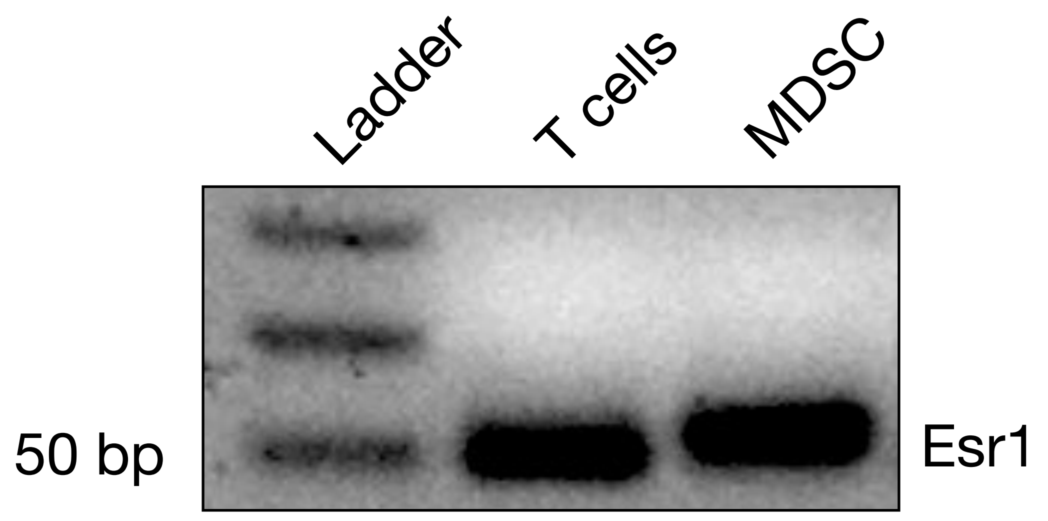

Supplementary Figure 3. Liver metastases-infiltrating T cells and MDSC express ERα
Fluorescence-activated cell sorting was performed on immune cells isolated 7 days post intrasplenic/portal injection of $5x10^5$ MC-38 cells that were immunostained with an anti-CD3 antibody (T cells), and with anti-CD11b, Ly6G and Ly6C antibodies (MDSC). PCR was performed on isolated immune cell RNA with Esr1 (ERα) primers.

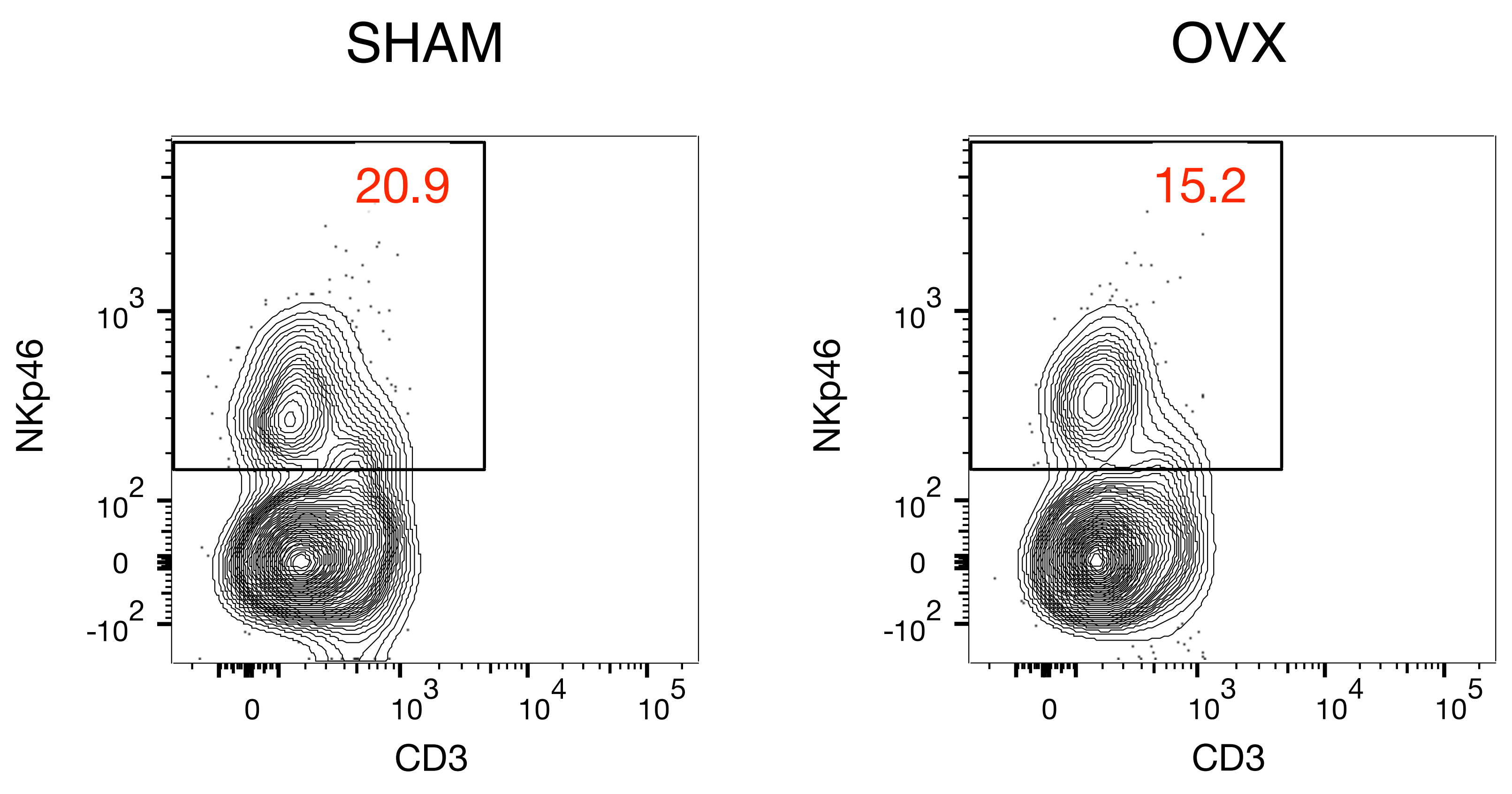

Supplementary Figure 4. Ovariectomy does not affect NK cell accumulation in the liver
Flow cytometry was performed on immune cells isolated 7 days post intrasplenic/portal injection of 5x10^5 MC-38 cells and immunostained with the indicated antibodies. Shown are flow cytometric contour plots obtained with the indicated NK cell population, identified as CD3$^-$NKp46$^+$. Hepatic immune cells were pooled from 5 livers per group.

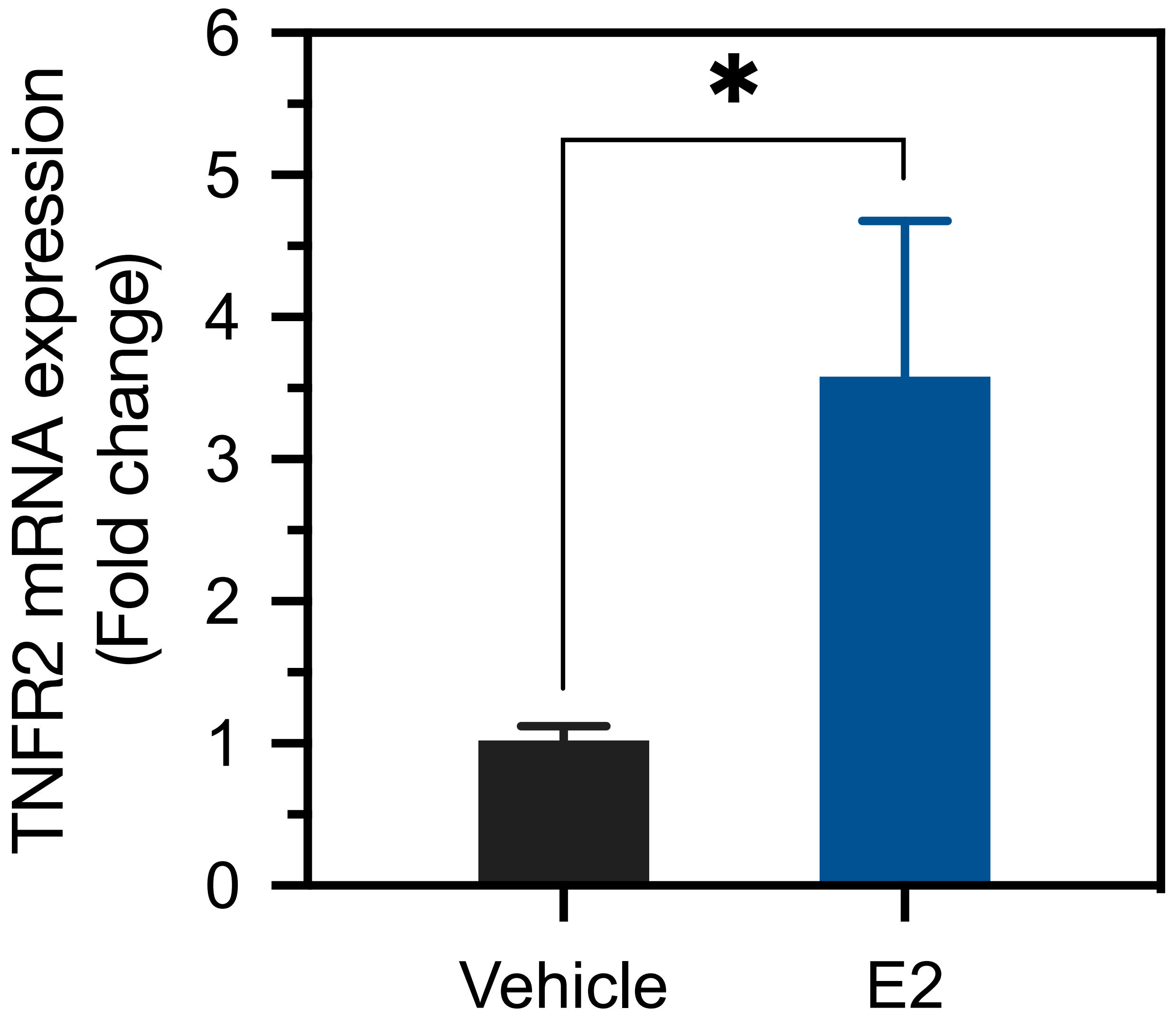

Supplementary Figure 5. Estradiol treatment increases TNFR2 expression in mouse splenocytes
Splenocytes were isolated from C57BL/6 mice, incubated *in vitro* for 6 hours in 10% FBS DMEM supplemented with 10^{-7} M β-estradiol or vehicle (0.01% ethyl alcohol) and their RNA extracted and analyzed by qPCR. Shown are results of qRT-PCR performed in triplicates with TNFR2 primers, normalized to GAPDH and expressed as mean (±SD) fold change relative to unstimulated cells that were assigned a value of 1. *-$p \leq 0.05$ as determined by the Student T test.

A

IDO1 Promoter

NC_000008.11:39927790-39928790 *Homo sapiens* chromosome 8, GRCh38.p7 Primary Assembly

```
GCTGTGACCTCCGTATTTCCTCTTTCTCTTTTTCCTATAGGACATGCTGCTCAGTTCCTCCAGGACATGA
GAAGATATATGCCACCAGCTCACAGGAACTTCCTGTGCTCATTAGAGTCAAATCCCTCAGTCCGTGAGTT
TGTCCTTTCAAAAGGTGATGCTGGCCTGCGGGAAGCTTATGACGCCTGTGTGAAAGCTCTGGTCTCCCTG
AGGAGCTACCATCTGCAAATCGTGACTAAGTACATCCTGATTCCTGCAAGCCAGCAGCCAAAGGAGAATA
AGACCTCTGAAGACCCTTCAAAACTGGAAGCCAAAGGAACTGGAGGCACTGATTTAATGAATTTCCTGAA
GACTGTAAGAAGTACAACTGAGAAATCCCTTTTGAAGGAAGGTTAATGTAACCCAACAAGAGCACATTTT
ATCATAGCAGAGACATCTGTATGCATTCCTGTCATTACCCATTGTAACAGAGCCACAAACTAATACTATG
CAATGTTTTACCAATAATGCAATACAAAAGACCTCAAAATACCTGTGCATTTCTTGTAGGAAAACAACAA
AAGGTAATTATGTGTAATTATACTAGAAGTTTTGTAATCTGTATCTTATCATTGGAATAAAATGACATTC
AATAAATAAAAATGCATAAGATATATTCTGTCGGCTGGGCGCGGTGGCTCACGCCTGTAATCCCAGCACT
TTGGGAGGCCGAGGCGGGCGGATCACAAGGTCAGGAGATCGAGACCATCTTGGCTAACACGGTGAAACCC
CGTCTCTACTAAAAATACAAAAAATTAGCCGGGCGCGGTGGCGGGCACCTGTAGTCCCAGCTACTCGGGA
GGCTGAGGCAGGAGAATGGCGTGAACCTGGGAGGCGGAGCTTGCAGTGAGCCAAGATTGTGCCACTGCAA
TCCGGCCTGGGCTAAAGAGCGGGACTCCGTCTCAAAAAAAAAAAAAAAAAAGATATATTCTGTCATAATAA
ATAAAAATGCATAAGATATAA
```

IDO2 Promoter

NC_000008.11:39933955-39934955 *Homo sapiens* chromosome 8, GRCh38.p7 Primary Assembly

```
GAAAATGATTCTTTCTGTTTGAAAAGTTGAGGAATGTTTTTTACAGGAGGTAAGTTTGATACTTAACTTT
AAAGTTGAATTGATTGGTGTACCTTCATTGAAAATTTTCTCCTGAATGCCCAGCATTAAATAAATAAGCA
AACATATAAGTTGCTTGTTGACCTAGCCTCATATATAATGCAAGAAGTAAACATAAACATATAAATGTTG
TAAATGAGCATCAGAATCATCTAATATATAGAATCTTCTGGGAGAGCTACTTTTTAAAACTATAAATTAA
TGATGATAGCTAATAATTACTTAATTCTTTCATATGCCTCTTACTGTCCAAAAAGTGTCATATACATTAA
TCACTTGATCAAAACAATGAATCTAGAGACAGGTACTATTACTAGTACTTCTTTTTCCTATATGGGAATT
GATACCCAATATAGTTCAGTAGTTTTCAAGAGCCACACAGCTATGACAAAGTAAAAGATTGAACCCAGGC
AATCTGACTGCAGAATACTTCAAACCGCTACACAATACAATCTCCTCTCTTGGAAAATGTTCTTTTACAA
GGGAACTAGGTTCCTTTTGAACTTTGCTATCACAGTATTGGATAATTGACTGCGCAAGTCTCTCCTCAAA
TTCCCAACTCATGCATGACTTTTTATTACGTTGTAGCAATAAAAGAGCAGTGGAGGTGGGGTGGAAGAAG
ACTCATTTTTGTCTTCATTACTGCTATGTGGAGCAAGGGCCCAAGCTTTTAAAAACAAGAGCTCAGAGAC
ATGGCCCTGGCCTTGGAAGATGCCCTAGAGACGCTGAGGTGGTTGTACTTTTGCCATAGGAGTGGCAGCC
AGAGAACTGAGCCCAATGAATGCAAAGGCTGGTGCCTGGAAATATTGTGACTTTGCCACAGAAAGAAGAT
GGAGAATTTTAAAGTTGGAAATCTGCCTGGTAAGGGATCATTTGCTGGTGTCTGCAAAGTTGAGTCCATA
CACACTGGTTTGGAAATTTCA
```

TNFRSF1b Promoter

NC_000001.11:12165948-12166948 *Homo sapiens* chromosome 1, GRCh38.p7 Primary Assembly

```
CCTGGTCTCAGCAGCCCCTCAGGGCCCTGCAGCTTCCCTGGTGACATTCTCTCCCAGCCTCTGTTCATCT
GCCCCCTGCCTGGGCAGGAGACTTGAGCAGGGAAGTGCAGAGTCTTCTCCCTGTGAGAAGGCTGGATGCG
TGTTTAAGGATAAATGAACACGCGAAGAGTAGTAACAACAGCCAAGATTTATAAATGCCTATTGTTATAT
ATGTAGATACTTACTTAAGTATATATAAAGTACAGACTGCATTATGTATATTACATATCTTTAAATTTTT
AGAATAGTCCTATGAGGTCAGTTCAGAGATCCAGACCCAGGTGGTCTGGCTCTAGAGTCTAAACAGGCCG
AGTGCAGTGGCTCACACCTATAATCCCAGCACCTTGGGAGGCCAGAGGCGGGAAGATCACTTGAGGGTGG
GAAGAACACGTGAGCTCAGGAGTTCGAGACCAGCCTGGACAACATGGCGAAACCCCATCTCTATAAAGAA
ATCAGCCTAGCATGGTGGCCCGAGCCTGTAGTCCCAGCTACTCGGGAGGCTGAGGTGGGAGGATCGCTTG
AGCGCAGGAGTTGGAGGCTGCAGTGAGCTATGGGTGAAAGAGTGAGACCTTGTCTCAAAAAAAATTAAAA
AATAAGAATTAAATATATTTAAAATAGAGTCTAAATAAGTGAATGATCTAGAATTCTCTTGGTTCCCCTA
AAGCAGCTGTCAGCTTTGGGGGATGTTTTTCCAAATTAGTGCCTCACCCTCACGGGACAGGGAAGCCTGT
GGGAGCTGGGAGGGCAGGTGGAGGCCGGGCAGGTGGAGGCCGGGCAGGTGGAGATGGTGATTCGAAGAGG
AGGGGACGATAGGAGGAGGTTGAGGGGTCACCCGAGTGCTGGGAGTGACGCTGGAGGTATCGGCCCAGCG
ATGCTGGAGTGGTCGGGTCGGAGGCCCAGCAGCGTCTGGGGAGGGGCGTGGGGGAGGCGTGTCCAAGGCC
GGCTGGCCCCGCCCCGCCCCG
```

B

IDO1 Promoter

NC_000074.6:c24584133-24583133 *Mus musculus* strain C57BL/6J chromosome 8, GRCm38.p4 C57BL/6J

```
AACTCTTGTGTCCTTTGTAGCGATAATTAAAGATTCCTCCAATGGATATCTACTTACCAGGGCTGAGTCC
TGTAGTTCCAAAATATTTATACTATGCTTTCTCATCTTGACTATCTCGTTAGTCTGAATTATCAACACAC
AAGAAGCTTTGGGCGCCAGACTATTGTTTGCCAGCCTTAGCAAATATCTCAGGAATGGATATGTAAAAGA
ATAAATGAGTTGTGAAACCAATCATGGGGTGAAGAACAATGAAGAGTGCACCTTTGTGGTGGTCTGAATG
AGGACTGTCCCCCATAGGTTCCCATATCTGGACACTTGCTTGCTGGGTGGTGGCACCATTTAGGGGTGGT
TATGGACTTTAGACAGTGTAGCTTTGCTGTCTGAAGTGGACTTTGAAGGTATAGAGCCTTGTCTTACTTG
CAGTTGACCGTGGTTAAACCAGTCAGCTTTTTGCTTCGGCTGCCTGCTTCAATGCCTTCCCAACTCTTAC
AGACACCCTCTCTGGGGACATAAGCCCCAATAAACTATTTTTTACATAAGTGGCTTCAGGTCATGGCACT
TCACCACAGAAACAGAAAAGTAATAATCTCCATAATGCAGCTTCTCCTGAGAGCCTGGGTTATTGGCACT
CACACTCTAACATGAGTTGATTTCATATGGGATCCTGTAGTGGTAAGTCAAGAGGAATTGAAGCAGAGTA
TAGTGGCTTGTGCCTGTAATCCCAGTGCAATGGCACAGAGCTAACACTCCAATACCAAACTCTGAAGCTC
CATGGCCTGCGTCCTGATTTTGTTCTGGGACTGAGGGTGCTGTAAAATAAAATAGTCTTTTATAGAGAAA
ATAGTTATTAATTTCCTTAACCTACATGATCTGCTTTGTACATCTAAACCCATTAGCAATGATTTGCCTT
CCGACACAAAGCAGATTCAAGCATACAGCAGGTCTTTAAGTCGTATTTATTCAAATGGACAGGTTATGTT
ACTTATATTAGAAGTCCTAAC
```

IDO2 Promoter

NC_000074.6:c24531892-24530892 *Mus musculus* strain C57BL/6J chromosome 8, GRCm38.p4 C57BL/6J

```
ACATTTCTAGCTCAAAGTCTTATTTATTCTTCCTGGCTGCAGAAAACGCCAGTCCCACCAGTTTAAAGTA
AATTCCATCCATTAGGGTTTCCCCACTTTGCAAATTCACATGTGTATTTGTGAATACACACCCGCAATGG
ACTCTCATTTCACGGTCCTTTCTAGGAGTATAGCATCAGCGAGGGCATCTGCTCATTGCCTGGCTTCTTC
CAAATGCTTCTCTCCTGAATGGAGCCTGGTAAGGTGGACTTAGCAAGCTGGTTCATCCTACCCAGAAAGA
CTTTCAGTCCCTTGGCCCTGCTGTTCTACAGTCCCTGTGACCATAGGGAGTACGAGACTGGCTTAGGTCC
TCTGCTTTCCAGGGGCTGAGGGAGAGAGAGAAATCTTAACTCCAAGAGAAAAATCAGCCAGGTCCTATAC
CAAAACTTCGGGGGCTCTTCCTGTCTTGTATCCTCTGGCCTCCATCTAGCATACGGGAGGTACAGTGACC
AGGCTAAGGGTTTCTCAGAGACCCGGTGCCACGTTCCTCTGTCTCTGCTGTGGCTGGGGTAGGTTCAACA
GTGACCACCAGGAGCTTACGATAATTCAGAAAGCCTTTCCTCCCCACCCGACGACATCCTTTTTGCCTGT
GGACAAAAGGCACCTCAGCAGAAGGGCACAGGTGTGCTTATGTACTCAAATCTTCTCTGGGATGAACCAC
ATGTGTGAGGGTCTAGAAAGTGTCTGGGATGAAGTCTAGTTACCTCCATGCCCTTGCTTTTCTCAGAGAG
TGGGGCCTTCGAGATAAGACCTGGCTTCCAATTCTATTGGGTCTCAGTTCTTGAAACAATATGGCTGCTG
GGCAGTGGTGGCACGTGCCTTTAATCCCAGAACTTGGGAGGCAGAGGCAGGAGGATTTCTGAGTTTGAGG
CCAGCCTGGTCTACAGAGTGAGTTCTAGGACAGCCAGGGCTACACAGAGAAACCCTGTCTCGAAGAAACC
AAAAAAAGAAAAAAAAAAGAAA
```

TNFRSF1b Promoter

NC_000070.6:c145212368-145211368 *Mus musculus* strain C57BL/6J chromosome 4, GRCm38.p4 C57BL/6J

```
CTGCCAGGTGTGGTAAACGTCTTCAATCCCAGCACTTAGAAAGCAGAGGCAGCTGGATCTCGGTGGTTTA
GAGGCCAGACTTGTCTCACAGTGAGTTCCAGGCCAGCCAAGACTACATAGTGAGTCCCTGACTTCAAATA
CATATAGCATTCTTGAGGCCAGAGAGCAGGCTTGGAGGATAAAGGGCCTTTCTGTTGAGGTTGGCAGCCT
TTGTTTGGTCTCATGTCCCACATGATGGAAGGACAGAGCTGACTGACCTTCACTCATACACTCAACACAC
ATACATAATAAACATAATTAATTTATTTAAAAAAAATATGTAGTATCCTTGGCTCAAACTGGAGCTCTTT
TGGTAAAGTGTTTGTCTAGCATGCCCAAGGCAGTGGGTTCAATCCCCACCACTGCATTTACTGGGTAGCC
TGTAATTCCAGCACTAGGAGGCAGAGGCAAAACAATCGAAAGTCAGTCATCCTTGGTTACAGGCCAGTTC
AGAGACAGCTCGGGGTACCTGAGAACTTGCCTCTGAAAGGAGGAGGAGGAAGAGAGAAGGAGAAAAGAAG
AAGAAGAAGAAGAAAAAGACAACACTAACATTGGTTTTGTTGTTGTTGTTCTGTTTGGTCTTTTTTT
TTGTTTTGGTTTTGTTTTGGTTTTTTGAGACAGGGTTTCTCTGTGTAGCCGTGGCTGTCCTGGAGCTCAC
TCCGTAGACCAGGCTGGCCTCAGACTCAGAAATCCTCCTGCCTCTGCCCGCTAAGTGCTGGGATTAAAGG
CATGTGCCACCACTGCCCGGCTAGCATTGTTCTTTTGAGGAGACCTTTTATAGATCATCTGAGAGTTAGT
GTAAGCTTTGTTTTAGAACATTGCAACATTGTATTAAAGCCGGACTCACAGATATTTAAGTGTTGCCTTA
GTTACTTTCCTCTTTACAATGAGGAAATACACAACAAAAGCAACCTAAGGAAGAAAGCATTTTAGGTTTT
GGTTTTGGCTTTGGTGTTGGC
```

Supplementary Figure 6. Partial estrogen response elements found upstream of the IDO1, IDO2 and TNFR2 transcription start sites.
Shown are 1 kb sequences upstream of the transcription start sites for the indicated human (A) and mouse (B) genes based on the NCBI website (open access). FASTA files were obtained and a putative transcription factor binding site analysis was performed using the ALGGEN PROMO online software to identify potential ERα and ERβ binding sites [1, 2].

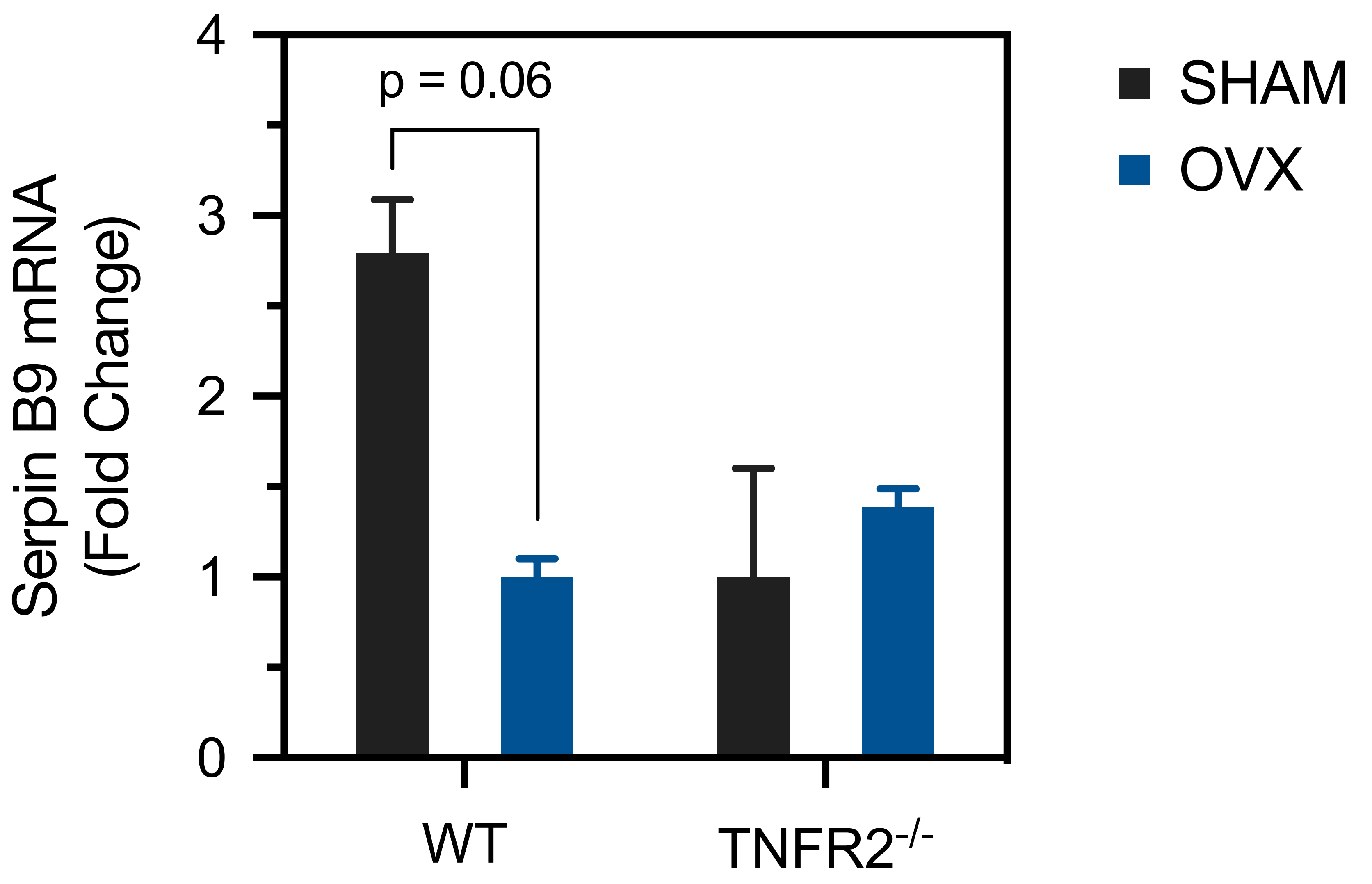

Supplementary Figure 7. The expression of Serpin B9 is not affected by ovariectomy in TNFR2-/- mice
Shown are results of qRT-PCR performed on whole liver RNA obtained from tumor-bearing WT or TNFR2-/- sham or OVX mice,. The results are based on livers obtained from 3 individual mice per group and expressed as means (± SD) relative to sham operated mice that were assigned a value of 1 all normalized to GAPDH. P value determined by the Student T test.

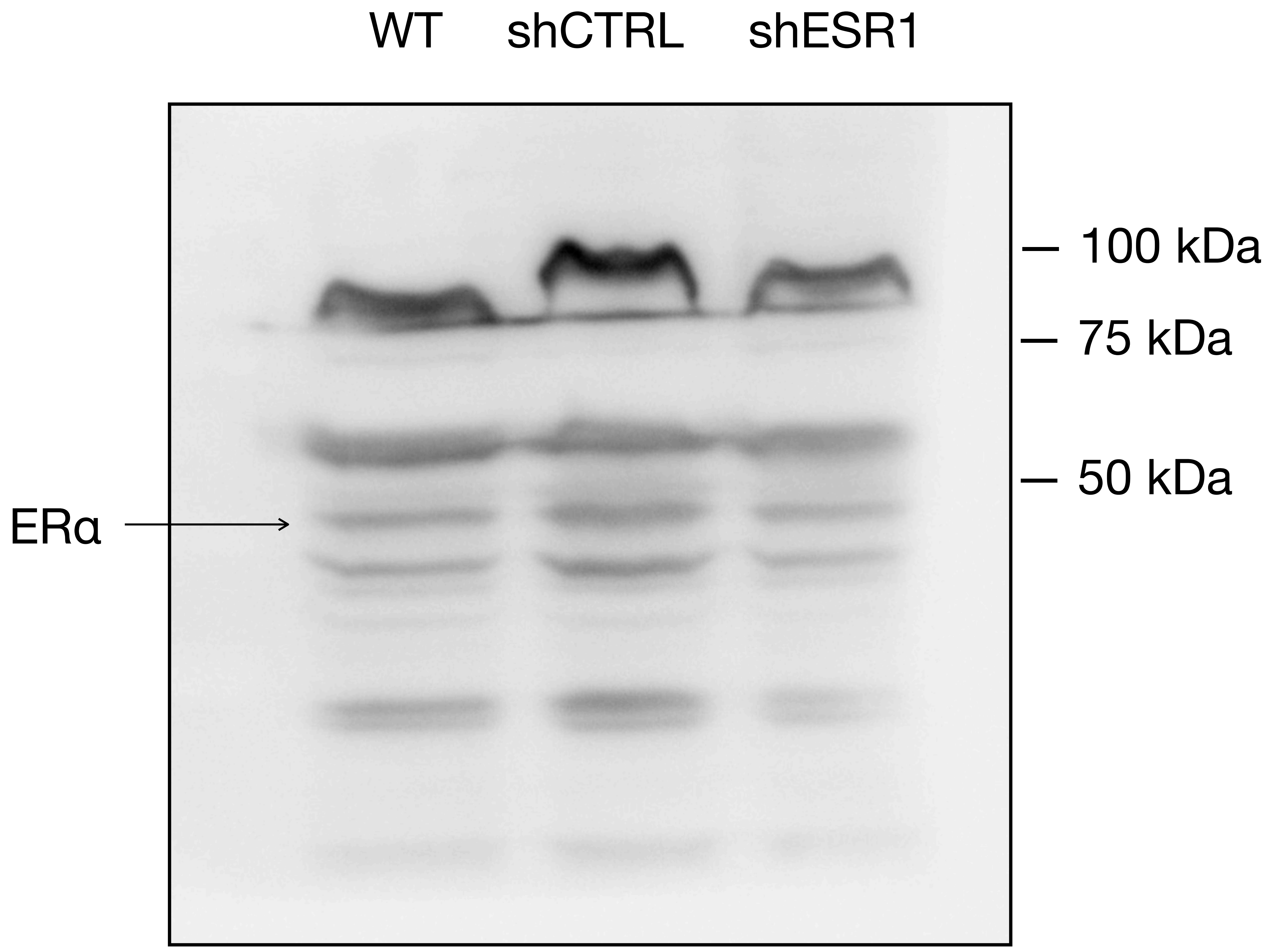

Supplementary Figure 8. *ESR1* knock-down in MC-38 colon carcinoma cells
Shown is a representative immunoblot of MC-38 cells stably transfected with a lentivirus expressing Esr1 shRNA (shESR1) or a scrambled sequence (shCTRL).

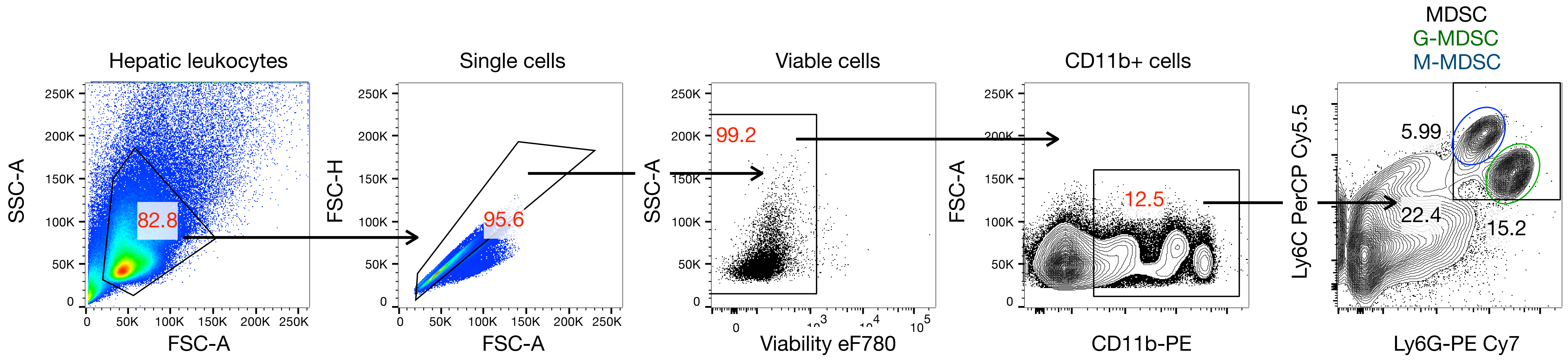

Supplementary Figure 9. Complete flow cytometry gating strategy for liver MDSC
Shown is a complete flow cytometric gating strategy for immunophenotyping and sorting hepatic MDSC

Supplementary Table 1:

Primers and Antibodies

qPCR Primer Sequences

Gene	Sequence Forward	Sequence Reverse
Esr1	TCTGCCAAGGAGACTCGCTA	GGTGCATTGGTTTGTAGCTG
GAPDH	ACCCAGAAGACTGTGGATGG	ACACATTGGGGGTAGGAACA
GrzB	CCTCCTGCTACTGCTGAC	GTCAGCACAAAGTCCTCTC
IDO1	CCCACACTGAGCACGGACGG	TTGCGGGGCAGCACCTTTCG
IDO2	GCCCAGAGCTCCGTGCTTCA	CAATCCAGCCATGCCTGTGG
SerpinB9	TATCTCAGGCACTTGGTTTG	TTTCTGTCTGGCTTGTTCAG
STAT3	TGATCGTGACTGAGGAGCTG	TGGCGGCTTAGTGAAGAAGT
TDO	GCCTTCCGTGTTCCTACCC	CAGTGGGCCCTCAGATGC

Flow Cytometry Antibodies

Name	Company	Cat. Number	Dilution
**CD11b** PE	BD Pharmigen	557397	1/1000
**CD11c** PE-Cy5.5	eBioscience	35-0114-82	1/100
**CD25** PE-Cy7	eBioscience	25-0251-82	1/100
**CD3e** APC	BD Pharmigen	553066	1/100
**CD4** FITC	eBioscience	11-0041-82	1/100
**CD8a** BV650	BD Horizon	563234	1/100
**Foxp3** eF610	Invitrogen	4332195	1/75
**IFN-γ** PE	BD Pharmigen	554412	1/75
**Ly6C** PerCP-Cy5.5	eBioscience	45-5932-80	1/1000
**Ly6G** PE-Cy7	eBioscience	25-5931-82	1/1000
**MHC II (I-A/I-E)** AF700	eBioscience	56-5321-82	1/100
**NKp46** eF610	eBioscience	61-3351-80	1/100
**TNFR2** BV421	BD Horizon	564088	1/100

Western Blot Antibodies

Name	Company	Cat. Number	Dilution
ER alpha	Invitrogen	PA5-16440	1/500
Beta actin	Sigma	A5441	1/2000
HRP-conjugated secondary Ab	Jackson ImmunoResearch Laboratories		1/5000

References for supplementary materials

1. Messeguer X, Escudero R, Farre D, Nunez O, Martinez J, Alba MM. PROMO: detection of known transcription regulatory elements using species-tailored searches. Bioinformatics 18, 333-334 (2002).

2. Farre D, et al. Identification of patterns in biological sequences at the ALGGEN server: PROMO and MALGEN. Nucleic Acids Res 31, 3651-3653 (2003).
