# Supplementary material for: Sexual dimorphism and the role of estrogen in the immune microenvironment of liver metastases
Source: Nat Commun. 2019 Dec 17;10:5745. doi: 10.1038/s41467-019-13571-x (PMC6917725; doi:10.1038/s41467-019-13571-x)
Supplement: Supplementary file 2 — Reporting Summary [file 41467_2019_13571_MOESM2_ESM.pdf]

nature research

Corresponding author(s): Pnina Brodt
Last updated by author(s): Oct 27, 2019

Reporting Summary

Nature Research wishes to improve the reproducibility of the work that we publish. This form provides structure for consistency and transparency in reporting. For further information on Nature Research policies, see Authors & Referees and the Editorial Policy Checklist.

Statistics

For all statistical analyses, confirm that the following items are present in the figure legend, table legend, main text, or Methods section.

n/a	Confirmed	
☐	☒	The exact sample size (n) for each experimental group/condition, given as a discrete number and unit of measurement
☐	☒	A statement on whether measurements were taken from distinct samples or whether the same sample was measured repeatedly
☐	☒	The statistical test(s) used AND whether they are one- or two-sided<br>*Only common tests should be described solely by name; describe more complex techniques in the Methods section.*
☐	☒	A description of all covariates tested
☐	☒	A description of any assumptions or corrections, such as tests of normality and adjustment for multiple comparisons
☐	☒	A full description of the statistical parameters including central tendency (e.g. means) or other basic estimates (e.g. regression coefficient) AND variation (e.g. standard deviation) or associated estimates of uncertainty (e.g. confidence intervals)
☒	☐	For null hypothesis testing, the test statistic (e.g. F, t, r) with confidence intervals, effect sizes, degrees of freedom and P value noted<br>*Give P values as exact values whenever suitable.*
☒	☐	For Bayesian analysis, information on the choice of priors and Markov chain Monte Carlo settings
☒	☐	For hierarchical and complex designs, identification of the appropriate level for tests and full reporting of outcomes
☒	☐	Estimates of effect sizes (e.g. Cohen's d, Pearson's r), indicating how they were calculated

Our web collection on statistics for biologists contains articles on many of the points above.

Software and code

Policy information about availability of computer code

Data collection	No software used
Data analysis	No software used

For manuscripts utilizing custom algorithms or software that are central to the research but not yet described in published literature, software must be made available to editors/reviewers. We strongly encourage code deposition in a community repository (e.g. GitHub). See the Nature Research guidelines for submitting code & software for further information.

Data

Policy information about availability of data

All manuscripts must include a data availability statement. This statement should provide the following information, where applicable:
- Accession codes, unique identifiers, or web links for publicly available datasets
- A list of figures that have associated raw data
- A description of any restrictions on data availability

The authors declare that the data supporting the findings of this study are available within the paper and its supplementary information files. All but one figure have associated raw data. Unprocessed (raw) data are available from the corresponding author upon reasonable request.

Field-specific reporting

Please select the one below that is the best fit for your research. If you are not sure, read the appropriate sections before making your selection.

☒ Life sciences ☐ Behavioural & social sciences ☐ Ecological, evolutionary & environmental sciences

Life sciences study design

All studies must disclose on these points even when the disclosure is negative.

Sample size	For experiments where data were reproducible, an n=3 was used to calculate means SD or SE. In some experiments cells were pooled from several mice to obtain adequate yields and the data presented as averages. This is stated in the manuscript, where applicable. Animal (metastasis) experiments involved at least 5 mice per group based on our previous experience for achieving statistical significance. Some in vivo experiemnts were repeated up to 3 times and data pooled.
Data exclusions	Relevant data were not excluded.
Replication	We use n=3 to verify reproducibility. For immunohistochemistry multiple images were analyzed as indicated.
Randomization	For all animal experiments, age-and sex-matched animals were used. Where treatment was applicable (i.e. Tamoxifen injection) mice were selected randomly and in an unbiased fashion for treatment or control groups.
Blinding	IHC analyses were performed blinded. Blinding is not applicable in FC and in vivo experiments.

Reporting for specific materials, systems and methods

We require information from authors about some types of materials, experimental systems and methods used in many studies. Here, indicate whether each material, system or method listed is relevant to your study. If you are not sure if a list item applies to your research, read the appropriate section before selecting a response.

Materials & experimental systems

n/a	Involved in the study	
☐	☒	Antibodies
☐	☒	Eukaryotic cell lines
☐	☐	Palaeontology
☐	☒	Animals and other organisms
☐	☐	Human research participants
☐	☐	Clinical data

Methods

n/a	Involved in the study	
☐	☐	ChIP-seq
☐	☒	Flow cytometry
☐	☐	MRI-based neuroimaging

Antibodies

Antibodies used	A list of all antibodies used, including manufacturer and catalog number, can be found in the supplementary info.
Validation	Antibodies for flow cytometry were selected based on recommendations by the Immunophenotyping Platform of the MUHC RI. Other antibodies were selected based on manufacturer's recommendation and published data (e.g., CD4-FITC [eBioscience, 11-041-82] has been validated in Teo et al, Nat Commun, 2018, doi: 10.1038/s41467-018-06227-9). All antibodies were optimized prior to use.

Eukaryotic cell lines

Policy information about cell lines

Cell line source(s)	MC-38 cells were originally from an NCI repository and were obtained as a kind gift from Dr. Shoshana Yakar (New York University, NY).<br>H-59 cells were generated in the Brodt laboratory and the LMP cells by the Lowy laboartory.
Authentication	MC-38 cells were recently authenticated by Didion and colleagues using SNP profiling.<br>H-59 cells were generated in the corresponding author's laboratory and maintained as a frozen stock. They are not cultured for more than 4 weeks prior to experiments to ensure authenticity. The LMP line was generated in the Lowy (co-author)'s laboratory and these cells were also maintained as a frozen stock to ensure phenotype stability.
Mycoplasma contamination	All cell lines used in this study tested negative for mycoplasma contamination as required by the policy of the MUHC RI.
Commonly misidentified lines<br>(See ICLAC register)	NA

Palaeontology

Specimen provenance	NA
Specimen deposition	NA
Dating methods	NA

☐ Tick this box to confirm that the raw and calibrated dates are available in the paper or in Supplementary Information.

Animals and other organisms

Policy information about studies involving animals; ARRIVE guidelines recommended for reporting animal research

Laboratory animals	C57Bl/6, BL6/129 F1 and nu/nu mice were used. Both female and male mice were used, as applicable. Mice were generally 7-12 weeks old when used in the experiments.
Wild animals	NA
Field-collected samples	NA
Ethics oversight	All mouse experiments were carried out in strict accordance with the guidelines of the Canadian Council on Animal Care (CCAC) ''Guide to the Care and Use of Experimental Animals'' and under the conditions and procedures approved by the Animal Care Committee of McGill University (AUP number: 5260)

Note that full information on the approval of the study protocol must also be provided in the manuscript.

Human research participants

Policy information about studies involving human research participants

Population characteristics	NA
Recruitment	NA
Ethics oversight	NA

Note that full information on the approval of the study protocol must also be provided in the manuscript.

Clinical data

Policy information about clinical studies

All manuscripts should comply with the ICMJE guidelines for publication of clinical research and a completed CONSORT checklist must be included with all submissions.

Clinical trial registration	NA
Study protocol	NA
Data collection	NA
Outcomes	NA

ChIP-seq

Data deposition

☐ Confirm that both raw and final processed data have been deposited in a public database such as GEO.

☐ Confirm that you have deposited or provided access to graph files (e.g. BED files) for the called peaks.

Data access links<br>*May remain private before publication.*	NA
Files in database submission	NA
Genome browser session<br>(e.g. UCSC)	NA

Methodology

Replicates	NA
Sequencing depth	NA
Antibodies	NA
Peak calling parameters	NA
Data quality	NA
Software	NA

Flow Cytometry

Plots

Confirm that:

☒ The axis labels state the marker and fluorochrome used (e.g. CD4-FITC).

☒ The axis scales are clearly visible. Include numbers along axes only for bottom left plot of group (a 'group' is an analysis of identical markers).

☒ All plots are contour plots with outliers or pseudocolor plots.

☒ A numerical value for number of cells or percentage (with statistics) is provided.

Methodology

Sample preparation	A detailed description of the immune cell isolation and staining protocol can be found in the Methods section
Instrument	BD LSRFortessa™ cell analyzer (BD Biosciences)
Software	FlowJo™ v10.6.1 (BD Biosciences)
Cell population abundance	NA
Gating strategy	For identifying MDSCs: FSC-A/SSC-A (hepatic leukocytes) > FSC-A/FSC-H (single cells) > eF780- (viable cells) > PE+ cells (CD11b+) > Ly6G+Ly6C+ cells (MDSC)<br>For identifying cytotoxic T cells: FSC-A/SSC-A (hepatic leukocytes) > FSC-A/FSC-H (single cells) > eF780- (viable cells) > APC+ cells (CD3+ T cells) > BV650+ cells (CD8+ CTL)

☒ Tick this box to confirm that a figure exemplifying the gating strategy is provided in the Supplementary Information.

Magnetic resonance imaging

Experimental design

Design type	NA
Design specifications	NA
Behavioral performance measures	NA

Acquisition

Imaging type(s)	NA
Field strength	NA
Sequence & imaging parameters	NA
Area of acquisition	NA

Diffusion MRI ☐ Used ☒ Not used

Preprocessing

Preprocessing software	NA

Normalization	NA
Normalization template	NA
Noise and artifact removal	*Describe your procedure(s) for artifact and structured noise removal, specifying motion parameters, tissue signals and physiological signals (heart rate, respiration).*
Volume censoring	NA

Statistical modeling & inference

Model type and settings	NA
Effect(s) tested	NA

Specify type of analysis: ☐ Whole brain ☐ ROI-based ☐ Both

Statistic type for inference (See Eklund et al. 2016)	NA
Correction	NA

Models & analysis

n/a	Involved in the study
☒	☐ Functional and/or effective connectivity
☒	☐ Graph analysis
☒	☐ Multivariate modeling or predictive analysis
